# Epidemiology of Cancer‐Associated Venous Thromboembolism Across the United States

**DOI:** 10.1002/ajh.70271

**Published:** 2026-03-08

**Authors:** Barbara D. Lam, Justine Ryu, Omid Jafari, Rock Bum Kim, Shengling Ma, Mrinal Ranjan, Jun Y. Jiang, Ang Li

**Affiliations:** ^1^ Division of Hematology & Oncology Fred Hutch Cancer Center, University of Washington Seattle Washington USA; ^2^ Department of Medicine Section of Hematology, Yale School of Medicine New Haven Connecticut USA; ^3^ Section of Hematology‐Oncology, Baylor College of Medicine Houston Texas USA

**Keywords:** cancer, thrombosis, venous thromboembolism

## Abstract

Prior epidemiological studies on cancer‐associated venous thromboembolism (VTE) were limited by homogenous patient populations. We leverage Cosmos, a collaborative dataset of Epic electronic health record systems, to conduct an updated evaluation of cancer‐associated VTE in the United States (US). Cosmos includes patients from all 50 states, and the dataset is representative of the US census in terms of age, race, ethnicity, and insurance coverage. We included patients in the US with a new cancer diagnosis between 2018 and 2023. We computed the cumulative incidence of VTE at 6 and 12 months after the diagnosis date and used Cox regression to identify risk factors and examine the association between VTE type within the first year and mortality. We identified 1 628 626 patients, with a cumulative incidence of VTE at 6 months of 2.7% and at 12 months of 3.7%. Major risk factors included prior VTE, cancer type, advanced stage, and chemotherapy‐based treatment regimens. The occurrence of VTE within the first year of cancer diagnosis was independently associated with increased mortality.

## 1 Introduction

Cancer greatly increases the risk of thrombosis, and it is estimated that up to 20% of patients with cancer will develop venous thromboembolism (VTE) during their disease course [1]. Cancer‐associated thrombosis (CAT) can interrupt or delay treatment plans and is a major driver of morbidity and mortality in patients with cancer [2]. The cancer treatment landscape is rapidly evolving with novel therapeutics and longer survival times, but the incidence of CAT has not decreased [2, 3, 4]. It is critical to continue reassessing CAT and its risk factors so that we can better understand how to prevent and treat thrombosis in cancer patients.

Recent epidemiological studies on CAT have been limited by the characteristics of their patient populations. A 2021 evaluation using the Danish Cancer Registry and Danish Patient Registry did not include data on race and ethnicity, limiting its generalizability to more diverse populations [4]. In the United States (US), a 2022 study using the California Cancer Registry included patients from only one state [5], while a 2023 study using the Veterans Affairs dataset was composed of 97% male patients [3].

Our study aims to evaluate the incidence of CAT using a dataset that is representative of the whole US. We utilize Epic Cosmos (cosmos.epic.com), a dataset created in collaboration with a community of Epic electronic health record (EHR) health systems. Cosmos integrates each patient's inpatient and outpatient charts across different health systems into one comprehensive record. It currently includes 300 million patients from four countries, most from the US. All states are represented and the database is comparable to the overall US census in terms of age, race, ethnicity, insurance coverage, and Social Vulnerability Index (SVI) [6].

Using this contemporary, longitudinal EHR dataset, we conducted a retrospective cohort study of patients with cancer in the US to characterize the incidence of CAT, how it has changed over time, and its association with cancer type, treatment, race, ethnicity, geographic region, and SVI, among other variables.

## 2 Methods

### 2.1 The Cosmos Dataset

We used retrospective data from Epic Cosmos. Cosmos is a live dataset, and current counts for patients, hospitals, and clinics are available on cosmos.epic.com. Epic generates the dataset by linking and deduplicating longitudinal patient‐level EHR data from participating health systems through the Care Everywhere network. HIPAA‐Defined Limited Data Sets are extracted every 2 weeks, curated and mapped to standard codes, and deidentified with removal of unstructured text and date‐shifting to produce the Expertly Determined De‐Identified (EDDI) Data Set. This study's analysis was performed using the 7/9/2025 EDDI data refresh.

### 2.2 Participants

For this study, we constructed a cohort of adults in Cosmos with newly diagnosed solid and hematologic neoplasms who were treated at eligible US health systems from 1/1/2018 to 12/31/2023. To minimize the risk of missing data as patients move between Cosmos and non‐Cosmos participating organizations, we only included organizations that contributed complete, continuous, cancer‐relevant EHR data across inpatient and outpatient settings (Table S1).

We further defined two active cohorts for analysis from the eligible sites. The primary cohort included all patients with newly diagnosed cancer (index date = date of cancer diagnosis). The secondary cohort included a subset who also received systemic therapy within 90 days after cancer diagnosis (index date = date of systemic therapy initiation).

**FIGURE 1:**
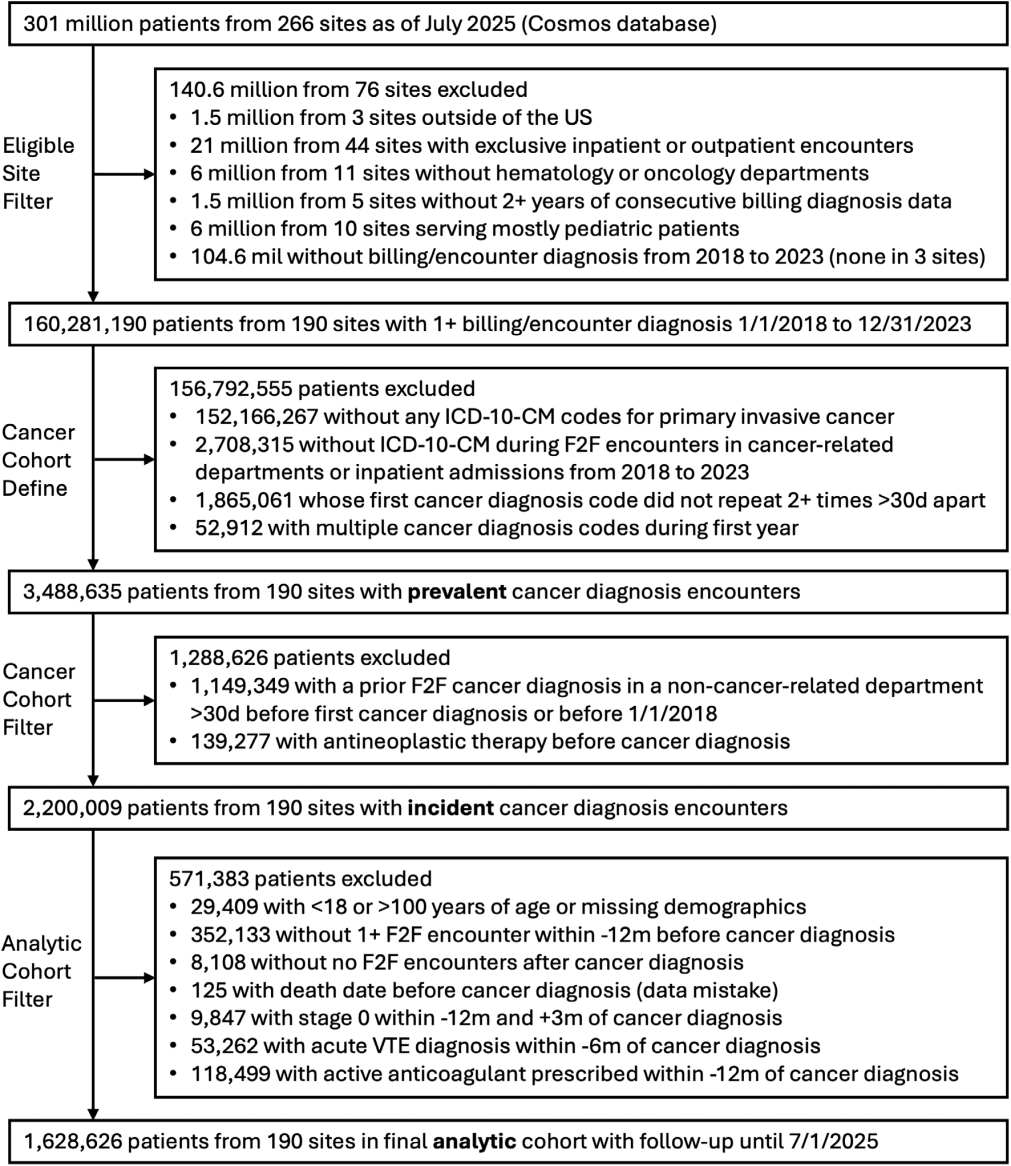
Patient selection diagram for study design and cohort exclusion.

To define newly diagnosed cancer (Figure 1), each patient had to have at least two invasive cancer *International Classification of Diseases, Tenth Edition, Clinical Modification* (ICD‐10‐CM) codes as billing final or encounter diagnoses > 30 days apart in selective face‐to‐face (F2F) encounters and department types during the study period (Table S2). Patients with non‐melanomatous skin cancers, multiple cancer diagnoses where the initial cancer diagnosis was different from the most commonly appearing one, premalignant conditions, unspecified or secondary metastatic ICD‐10‐CM codes without a primary site, or previous cancer diagnosis codes or systemic therapy receipt within the year prior to first cancer diagnosis (including in the year 2017) were excluded. To define new systemic therapy, we extracted newly prescribed or administered antineoplastic medications after cancer diagnosis date using the therapeutic classification from the First Databank drug database (Table S3). The medications were classified as cytotoxic chemotherapy, immune checkpoint inhibitor, targeted therapy, or endocrine therapy. Systemic therapy exposures were modeled as non‐hierarchical, time‐varying covariates. Receipt of therapy within 90 days of cancer diagnosis defined the treated cohort, while receipt within 365 days of diagnosis was used to model time‐varying treatment exposure in multivariable analyses.

For the final analytic cohort, we applied additional lookback and follow‐up exclusions from the index date of cancer diagnosis (Table S2). Additional exclusions included age < 18 or > 100 years, death prior to the index date, maximum cancer stage of 0, an active anticoagulant prescription at the index date, or a diagnosis of acute VTE within the prior 6 months.

### 2.3 Outcomes

To decrease interval missingness, the censor date was defined as the last F2F encounter before a 6‐month encounter‐free gap. Patients were followed until the earliest outcome date, censor date, death, or 7/9/2025.

The primary outcome was incident VTE, which was defined as the first occurrence of acute pulmonary embolism (PE), lower‐extremity deep vein thrombosis (LE‐DVT), or upper‐extremity deep vein thrombosis (UE‐DVT) (Table S4). We used our previously validated ICD‐10‐CM and medication‐based phenotype algorithm to define incident VTE using billing final diagnoses and encounter diagnoses from any inpatient F2F encounter or 2+ outpatient F2F encounters > 30 to < 365 days apart (positive predictive value [PPV] 95%) [7]. ICD codes for superficial venous thromboses, splanchnic vein thromboses, septic emboli, and other atypical site thromboses were excluded.

Secondary outcomes included death from any cause and its association with type and timing of VTE. The death date and last known follow‐up date were extracted directly from Cosmos.

### 2.4 Covariates

Baseline variables (Table S5) were identified based on clinical knowledge of VTE risk factors and included age; sex; race; ethnicity; primary language; marital status; state (grouped by region); Rural–Urban Commuting Area (RUCA) code; SVI; 26 consolidated cancer types; cancer stage; 4 systemic therapy types; 16 comorbidities (including paralysis) from the National Cancer Institute Comorbidity Index (NCI‐CI); height; weight; BMI; recent/current hospitalization of > 3 days; CBC including white blood cell (WBC), hemoglobin (Hb), platelet (Plt), alanine transaminase (ALT); total bilirubin (TB), creatinine, and estimated glomerular filtration rate (eGFR) based on the Cockcroft‐Gault formula; history of VTE (historic/chronic VTE at any time or acute VTE > 6 months before index date); and use of anticoagulant or antiplatelet medications. All laboratory features were defined using Logical Observation Identifiers, Names, and Codes (LOINC). Baseline laboratory covariates were defined using values measured closest to the cancer diagnosis date and were treated as fixed baseline covariates in all analyses, including those of the secondary cohort receiving systemic therapy. Due to significant missingness in reported cancer stage (~50%), we further defined metastatic disease using ICD‐10‐CM codes. Advanced stage was subsequently determined as stages III–IV or having a metastatic ICD code.

### 2.5 Statistical Analysis

Descriptive statistics were used to examine the distribution of categorical and continuous variables. All trend analyses were performed in the primary cancer cohort and the subset of patients receiving systemic therapy within the first 90 days after cancer diagnosis. VTE incidence was estimated using cumulative incidence functions with death as a competing risk. Anticoagulant prescription pattern was displayed as the proportion of individual drug divided by all anticoagulant prescriptions within 30 days of new VTE diagnosis for each study year.

We used multivariable Cox regression with a robust variance estimator clustered on individual patients to calculate hazard ratios (HRs) and assess associations between baseline variables and systemic therapy initiation (time‐varying covariate) and VTE risk within 12 months. Given the large cohort size, in which nearly all covariates would likely achieve statistical significance in univariate testing, we used cross‐validated Least Absolute Shrinkage and Selection Operator (LASSO) model to select features with both statistical robustness and clinical relevance.

For mortality analyses, we used multivariable Cox regression to examine the association between the type of VTE onset (time‐varying covariate) and 12‐month mortality after adjusting for confounders associated with death. All analyses were performed using R version 4.4.3 (R Foundation for Statistical Computing, Vienna, Austria). The study was deemed non‐human subject research by the Institutional Review Board at Baylor College of Medicine.

## 3 Results

### 3.1 Participants

As of 7/9/2025, there were 300 million unique patients from 256 sites in Cosmos. After applying exclusion criteria, our final cohort included 1 628 626 patients (858 935 women [52.7%]; median [IQR] age 66 [58–74] years from 173 healthcare systems) from 1/1/2018 to 12/31/2023 (Table 1, Table S6). The median (IQR) continuous follow‐up time was 682 (340–1124) days for VTE ascertainment and 1269 (724–1856) days for mortality. Most patients self‐identified as White (1 314 927; 80.7%), with 200 026 (12.3%) patients identifying as Black, 91 881 (5.6%) patients as Hispanic, and 52 852 (3.2%) patients as Asian/Pacific Islander. Most patients lived in a metropolitan area (1 308 212 [80.3%]) and in regions 4 (328 552 [20.2%]) and 5 (382 394 [23.5%]) of the US, as defined by the Department of Health and Human Services (HHS). There was a steady accrual of patients each year, with 14%–20% of patients incidentally diagnosed with cancer in each year between 2018–2023. The most common cancer types were breast (366 213 [22.5%]), prostate (272 572 [16.7%]), lung (151 947 [9.3%]), and colorectal (108 150 [6.6%]). Among patients with reported stage, 14.0% were metastatic at the time of diagnosis. Systemic therapy was given to 566 258 (34.8%) of patients within 3 months of cancer diagnosis.

**TABLE 1:** Baseline patient characteristics.

Characteristic	Overall *N* = 1 628 626	Treated within 90 days *N* = 562 110
Age		
Mean (SD)	64.9 (13.2)	63.8 (13.4)
Median (Q1, Q3)	66.0 (58.0, 74.0)	65.0 (56.0, 73.0)
Min, Max	18.0, 100.0	18.0, 100.0
Sex		
Female	858 935 (52.7%)	335 623 (59.7%)
Male	769 691 (47.3%)	226 487 (40.3%)
Race		
White	1 314 927 (80.7%)	450 500 (80.1%)
Black	200 026 (12.3%)	69 189 (12.3%)
Asian Pacific Islander	52 852 (3.2%)	20 855 (3.7%)
American Indian/Alaska Native	13 813 (0.8%)	4875 (0.9%)
Other	34 799 (2.1%)	12 781 (2.3%)
Unknown	12 209 (0.7%)	3910 (0.7%)
Ethnicity		
Non‐Hispanic	1 488 634 (91.4%)	512 854 (91.2%)
Hispanic	91 881 (5.6%)	33 466 (6.0%)
Unknown	48 111 (3.0%)	15 790 (2.8%)
Body mass index (BMI)		
Normal BMI	419 384 (25.8%)	157 228 (28.0%)
Underweight	35 741 (2.2%)	13 650 (2.4%)
Overweight (BMI 25–30)	529 340 (32.5%)	181 385 (32.3%)
Obesity I (BMI 30–35)	338 697 (20.8%)	114 839 (20.4%)
Obesity II (BMI 35–40)	157 592 (9.7%)	54 223 (9.6%)
Obesity III (BMI ≥ 40)	110 470 (6.8%)	36 888 (6.6%)
Missing	37 402 (2.3%)	3897 (0.7%)
State region (HHS classification)^a^		
Region 1	120 004 (7.4%)	38 484 (6.8%)
Region 2	116 539 (7.2%)	37 820 (6.7%)
Region 3	237 698 (14.6%)	78 200 (13.9%)
Region 4	328 552 (20.2%)	114 334 (20.3%)
Region 5	382 394 (23.5%)	135 120 (24.0%)
Region 6	144 766 (8.9%)	46 209 (8.2%)
Region 7	78 507 (4.8%)	30 109 (5.4%)
Region 8	66 114 (4.1%)	26 963 (4.8%)
Region 9	99 808 (6.1%)	32 791 (5.8%)
Region 10	54 042 (3.3%)	22 005 (3.9%)
Unknown	202 (0.0%)	75 (0.0%)
Social vulnerability index		
Mean (SD)	0.5 (0.3)	0.5 (0.3)
Median (Q1, Q3)	0.6 (0.3, 0.8)	0.6 (0.3, 0.8)
Min, Max	0.0, 1.0	0.0, 1.0
NCI comorbidity index		
Mean (SD)	0.3 (0.5)	0.3 (0.5)
Median (Q1, Q3)	0.0 (0.0, 0.5)	0.0 (0.0, 0.5)
Min, Max	0.0, 4.9	0.0, 4.1
Cancer group		
Breast	366 213 (22.5%)	181 259 (32.2%)
Prostate	272 572 (16.7%)	53 933 (9.6%)
Head and neck	70 163 (4.3%)	21 113 (3.8%)
Lung	151 947 (9.3%)	56 937 (10.1%)
Lower gastrointestinal	108 150 (6.6%)	40 643 (7.2%)
Upper gastrointestinal	29 675 (1.8%)	15 448 (2.7%)
Pancreas	34 047 (2.1%)	18 859 (3.4%)
Liver	19 164 (1.2%)	5018 (0.9%)
Bile and gallbladder	12 958 (0.8%)	5731 (1.0%)
Kidney	48 673 (3.0%)	5493 (1.0%)
Bladder	56 012 (3.4%)	8593 (1.5%)
Testis	10 076 (0.6%)	2810 (0.5%)
Cervix	15 621 (1.0%)	6118 (1.1%)
Ovary	21 301 (1.3%)	10 679 (1.9%)
Uterus	63 637 (3.9%)	11 492 (2.0%)
Central nervous system	22 245 (1.4%)	11 693 (2.1%)
Melanoma	39 580 (2.4%)	6999 (1.2%)
Sarcoma	18 342 (1.1%)	3284 (0.6%)
Myeloma	31 627 (1.9%)	18 342 (3.3%)
Acute myeloid leukemia	9699 (0.6%)	7306 (1.3%)
Acute lymphoblastic leukemia	2624 (0.2%)	1912 (0.3%)
Chronic myeloid leukemia or myelodysplastic syndrome	34 362 (2.1%)	10 615 (1.9%)
Chronic lymphocytic leukemia	35 670 (2.2%)	4572 (0.8%)
Aggressive lymphoma	31 214 (1.9%)	21 109 (3.8%)
Indolent lymphoma	38 724 (2.4%)	16 567 (2.9%)
Other cancer	84 330 (5.2%)	15 585 (2.8%)
Cancer stage		
Stage I	193 558 (11.9%)	76 941 (13.7%)
Stage II	94 185 (5.8%)	42 402 (7.5%)
Stage III	86 201 (5.3%)	53 022 (9.4%)
Stage IV/Metastatic	228 199 (14.0%)	131 946 (23.4%)
Unstageable^b^	190 921 (11.7%)	81 389 (14.5%)
Unknown	835 562 (51.3%)	176 410 (31.4%)
Systemic therapy within 365 days^c^		
Chemotherapy	376 422 (23.1%)	335 158 (59.6%)
Immune checkpoint inhibitor	60 685 (3.7%)	50 549 (9.0%)
Targeted therapy	136 489 (8.4%)	118 266 (21.0%)
Endocrine therapy	233 308 (14.3%)	164 145 (29.2%)
VTE and bleeding history		
VTE history last year (> 6 months)	44 865 (2.8%)	13 616 (2.4%)
Bleeding history last year	187 894 (11.5%)	63 819 (11.4%)

Abbreviations: HHS, health and human services; NCI, National Cancer Institute; Q, quantile; SD, standard deviation; VTE, venous thromboembolism.

^a^ Region 1 (Connecticut, Maine, Massachusetts, New Hampshire, Rhode Island, Vermont); Region 2 (New Jersey, New York, Puerto Rico, the Virgin Islands); Region 3 (Delaware, District of Columbia, Maryland, Pennsylvania, Virginia, West Virginia); Region 4 (Alabama, Florida, Georgia, Kentucky, Mississippi, North Carolina, South Carolina, Tennessee); Region 5 (Illinois, Indiana, Michigan, Minnesota, Ohio, Wisconsin); Region 6 (Arkansas, Louisiana, New Mexico, Oklahoma, Texas); Region 7 (Iowa, Kansas, Missouri, Nebraska); Region 8 (Colorado, Montana, North Dakota, South Dakota, Utah, Wyoming); Region 9 (Arizona, California, Hawaii, Nevada, American Samoa, Commonwealth of the Northern Mariana Islands, Federated States of Micronesia, Guam, Marshall Islands, Republic of Palau); Region 10 (Alaska, Idaho, Oregon, Washington).

^b^ Unstageable cancers included leukemias and primary brain tumors.

^c^ Treatment categories are not mutually exclusive and reflect the receipt of each individual treatment type within 1 year after cancer diagnosis.

### 3.2 VTE Incidence and Anticoagulant Use

**FIGURE 2:**
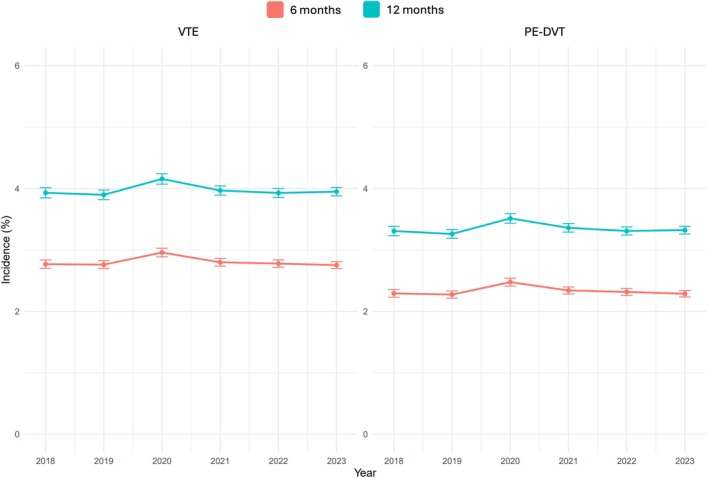
Cancer‐associated venous thromboembolism (VTE) incidence over time. [Color figure can be viewed at wileyonlinelibrary.com]

Before cancer diagnosis, 2.8% of patients had a remote VTE history and 3.0% had a VTE in the last 6 months. After excluding recent events before cancer diagnosis, the incidence of new VTE at 6 and 12 months after cancer diagnosis was 2.68% (95% confidence interval [CI] 2.66%–2.70%, *n* = 43 806) and 3.66% (95% CI, 3.63%–3.69%, *n* = 59 966), respectively. Notably, 66% of the incident VTE events were identified from the inpatient setting. In non‐mutually exclusive counts, there were 31 910 PE, 28 366 LE‐DVT, and 11 444 UE‐DVT events within the first 12 months. In the cohort receiving systemic therapy within 90 days (*n* = 566 258), the incidence of new VTE at 6 and 12 months after therapy was higher at 4.36% (95% CI, 4.30%–4.41%, *n* = 24 544) and 5.68% (95% CI, 5.62%–5.74%, *n* = 31 963), respectively. Yearly trends in VTE incidence remained unchanged through the 6 years (Figure 2).

**FIGURE 3:**
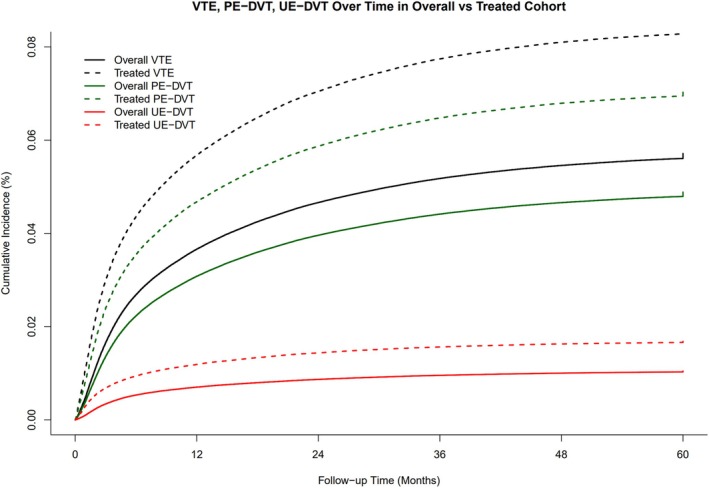
Cumulative incidence of venous thromboembolism in the overall cohort compared to the cohort of patients who received systemic therapy. [Color figure can be viewed at wileyonlinelibrary.com]

Cumulative incidence curves demonstrated higher rates of VTE in the treated cohort compared with the overall cancer cohort across all VTE subtypes (Figure 3). By 60 months, the cumulative incidence of VTE was 5.71% (95% CI, 5.68%–5.75%) in the overall cohort and 8.40% (95% CI, 8.33%–8.47%) in the treated cohort. For PE/LE‐DVT, incidence reached 4.89% (95% CI, 4.85%–4.92%) and 7.05% (95% CI, 6.99%–7.12%) respectively, while UE‐DVT incidence reached 1.05% (95% CI, 1.03%–1.06%) and 1.68% (95% CI, 1.65%–1.71%) respectively. In all subtypes, the risk of VTE rose steeply within the first few months after cancer diagnosis and then plateaued, with persistent separation between treated and untreated groups throughout follow‐up.

**FIGURE 4:**
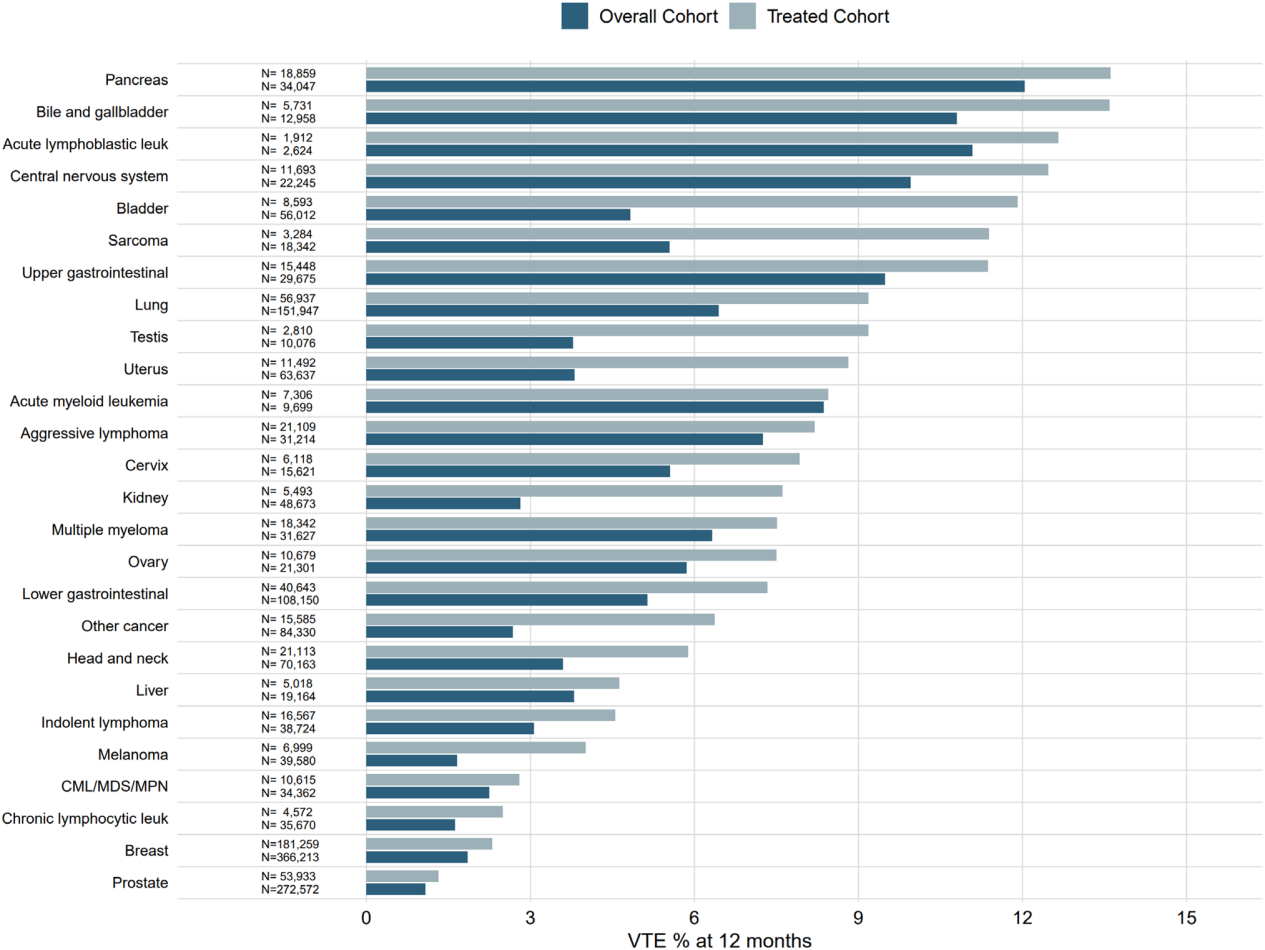
Cumulative incidence at 12 months, stratified by cancer type. (A) Overall cohort. (B) Treated cohort. [Color figure can be viewed at wileyonlinelibrary.com]

In both the overall and treated cohorts, patients with pancreatic cancer (12.0% [95% CI, 11.7%–12.4%], 13.6% [95% CI, 13.1%–14.1%]), bile and gallbladder cancer (10.8% [95% CI, 10.3%–11.3%], 13.6% [95% CI, 12.7%–14.5%]), and acute lymphoblastic leukemia (ALL) (11.1% [95% CI, 9.9%–12.3%], 12.7% [95% CI, 11.1%–14.1%]) had the highest incidence of VTE at 12 months (Figure 4). In the overall cohort, patients with melanoma (1.7% [95% CI, 1.5%–1.8%]), chronic lymphocytic leukemia (CLL) (1.6% [95% CI, 1.5%–1.8%]), and prostate cancer (1.1% [95% CI, 1.0%–1.1%]) had the lowest incidence of VTE at 12 months. In the treated cohort, patients with CLL (2.5% [95% CI, 2.0%–2.9%]), breast (2.3% [95% CI, 2.2%–2.4%]), and prostate cancer (1.3% [95% CI, 1.2%–1.4%]) had the lowest incidence of VTE at 12 months. VTE incidence exceeded 10% in three cancer types in the overall cohort and in seven cancer types in the treated cohort. In some cancer types, VTE incidence increased more than two‐fold when patients were treated, including in bladder (4.8% [95% CI, 4.7%–5.0%] to 11.9% [95% CI, 11.2%–12.6%]), sarcoma (5.5% [95% CI, 5.2%–5.9%] to 11.4% [95% CI, 1.03%–12.5%]), testis (3.8% [95% CI, 3.4%–4.2%] to 9.2% [95% CI, 8.1%–10.2%]), uterus (3.8% [95% CI, 3.7%–4.0%] to 8.8% [95% CI, 8.3%–9.3%]), kidney (2.8% [95% CI, 2.7%–3.0%] to 7.6% [95% CI, 6.9%–8.3%]), and melanoma (1.7% [95% CI, 1.5%–1.8%] to 4.0% [95% CI, 3.6%–4.5%]).

Patients with pancreatic cancer had the highest incidence of PE/LE‐DVT at 12 months in the overall cohort (10.5% [95% CI, 10.3%–10.9%]) while patients with central nervous system (CNS) cancer had the highest incidence of PE/LE‐DVT in the treated cohort (12.3% [95% CI, 11.7%–12.9%]) (Figure S1). Patients with ALL had the highest incidence of UE‐DVT at 12 months in both the overall cohort (5.9% [95% CI, 5.0%–6.8%]) and the treated cohort (6.7% [95% CI, 5.6%–7.8%]) (Figure S2). Cumulative incidence of overall VTE, PE/DVT‐PE, and UE‐DVT at 12 months stratified by cancer type can be found in Table S7.

**FIGURE 5:**
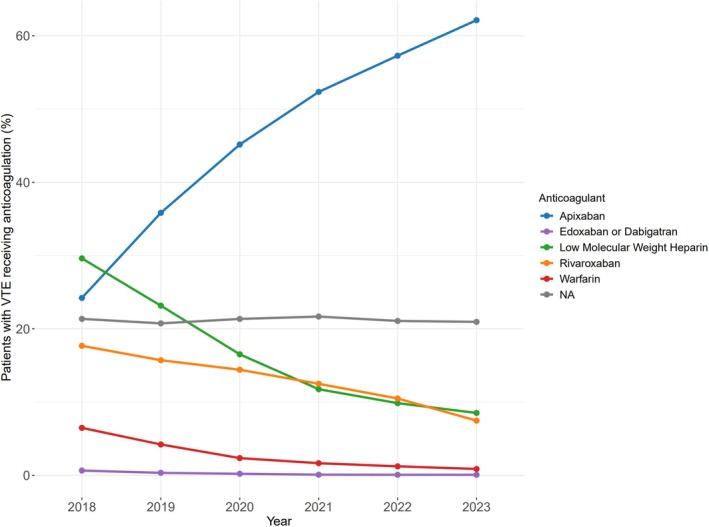
Trends in anticoagulation prescription over time. [Color figure can be viewed at wileyonlinelibrary.com]

Among patients who developed VTE, 79.0% received a therapeutic anticoagulant prescription within 30 days. The predominant type of anticoagulant prescription shifted over the study period (Figure 5). The proportion of apixaban prescriptions increased over time from 24.2% in 2018 to 62.1% in 2023, while use of other anticoagulants declined over the same period, most notably low molecular weight heparin, which decreased from 29.6% to 8.5% (Table S8).

### 3.3 Variables Associated With CAT

For the multivariable analysis, the following variables were selected after LASSO regression: age, sex, race, BMI, cancer type, cancer stage, cancer therapy, comorbidities, history of VTE, history of bleeding, WBC, Hb, Plt, and albumin. Cancer type and stage were most significantly associated with VTE risk. It was higher for patients with stage IV cancer (HR 3.17; 95% confidence interval [CI] 3.02–3.32) compared to those with stage I cancer, and risk increased steadily with stage (Table 2). Compared with breast cancer, pancreatic (HR 3.3; 95% CI 3.1–3.4), bile/gallbladder (HR 2.6; 95% CI 2.5–2.6), and upper gastrointestinal (GI) cancer (HR 2.5; 95% CI 2.4–2.6) were associated with the greatest risk of VTE.

**TABLE 2:** Multivariable Cox regression analysis for association with cancer‐associated thrombosis.

Characteristic	Hazard ratio	95% confidence interval	*p*
Age	1.09	1.09, 1.10	< 0.001
Sex			
Female	Ref	Ref	Ref
Male	1.06	1.04, 1.08	< 0.001
Race			
White	Ref	Ref	Ref
Black	1.35	1.32, 1.38	< 0.001
Asian Pacific Islander	0.72	0.68, 0.76	< 0.001
American Indian/Alaska Native	0.93	0.85, 1.02	0.13
Other	0.97	0.92, 1.03	0.3
Unknown	0.89	0.80, 1.00	0.046
Cancer type			
Breast	Ref	Ref	Ref
Prostate	0.65	0.62, 0.69	< 0.001
Head and neck	1.31	1.25, 1.38	< 0.001
Lung	1.85	1.78, 1.92	< 0.001
Lower gastrointestinal	1.39	1.33, 1.45	< 0.001
Upper gastrointestinal	2.49	2.37, 2.62	< 0.001
Pancreas	3.24	3.10, 3.39	< 0.001
Liver	1.42	1.32, 1.54	< 0.001
Bile and gallbladder	2.61	2.46, 2.78	< 0.001
Kidney	1.15	1.08, 1.23	< 0.001
Bladder	2.09	1.99, 2.20	< 0.001
Testis	2.11	1.89, 2.34	< 0.001
Cervix	1.95	1.81, 2.10	< 0.001
Ovary	1.35	1.27, 1.44	< 0.001
Uterus	1.47	1.40, 1.55	< 0.001
Central nervous system	2.42	2.25, 2.62	< 0.001
Melanoma	0.89	0.82, 0.96	0.004
Sarcoma	2.36	2.20, 2.53	< 0.001
Myeloma	1.31	1.22, 1.41	< 0.001
Acute myeloid leukemia	1.40	1.27, 1.55	< 0.001
Acute lymphoblastic leukemia	1.98	1.73, 2.26	< 0.001
Chronic myeloid leukemia or myelodysplastic syndrome	0.54	0.49, 0.60	< 0.001
Chronic lymphocytic leukemia	0.47	0.42, 0.52	< 0.001
Aggressive lymphoma	1.22	1.14, 1.32	< 0.001
Indolent lymphoma	0.74	0.68, 0.81	< 0.001
Other cancer	1.13	1.07, 1.18	< 0.001
Cancer stage			
Stage I	Ref	Ref	Ref
Stage II	1.67	1.59, 1.76	< 0.001
Stage III	2.13	2.03, 2.23	< 0.001
Stage IV	3.23	3.10, 3.37	< 0.001
Unstageable	2.31	2.15, 2.48	< 0.001
Unknown	1.45	1.39, 1.51	< 0.001
Treatment^a^			
Chemotherapy	1.93	1.90, 1.97	< 0.001
Immune checkpoint inhibitor	1.21	1.17, 1.25	< 0.001
Targeted therapy	1.2	1.16, 1.23	< 0.001
Endocrine therapy	0.78	0.75, 0.82	< 0.001
Medical history			
Prior hospitalization	1.23	1.20, 1.26	< 0.001
Prior paralysis	1.45	1.36, 1.54	< 0.001
Prior VTE	2.28	2.21, 2.36	< 0.001
Prior bleeding	1.04	1.02, 1.07	< 0.001
Body mass index (BMI)			
Normal BMI	Ref	Ref	Ref
Underweight	0.92	0.87, 0.97	0.003
Overweight (BMI 25–30)	1.16	1.13, 1.18	< 0.001
Obesity I (BMI 30–35)	1.37	1.34, 1.41	< 0.001
Obesity II (BMI 35–40)	1.52	1.48, 1.57	< 0.001
Obesity III (BMI ≥ 40)	1.78	1.72, 1.84	< 0.001
Missing	0.7	0.63, 0.78	< 0.001
Labs^b^			
White blood cell count ≤ 11	Ref	Ref	Ref
White blood cell count > 11	1.19	1.17, 1.22	< 0.001
Hemoglobin ≥ 10	Ref	Ref	Ref
Hemoglobin < 10	1.08	1.05, 1.11	< 0.001
Platelet count < 350	Ref	Ref	Ref
Platelet count ≥ 350	1.2	1.17, 1.23	< 0.001
Albumin ≥ 3.5	Ref	Ref	Ref
Albumin < 3.5	1.41	1.38, 1.44	< 0.001

Abbreviation: Ref, reference.

^a^ Time‐varying covariate within 1 year, categories are not mutually exclusive.

^b^ Missing lab values were modeled as a separate category and results are not shown.

Systemic treatment, age, sex, race, body mass index, hospitalization status, history of paralysis, history of VTE, WBC > 11 × 10^9^/L, Hb < 10 g/dL, Plt > 350 × 10^9^/L, and albumin < 3.5 g/dL were each independently associated with VTE risk. Compared with those untreated, patients receiving chemotherapy (HR 1.9; 95% CI, 1.9–1.9), immune checkpoint inhibitor (HR 1.2; 95% CI, 1.2–1.3), and targeted therapy (HR 1.2; 95% CI, 1.2–1.2) had a higher risk of VTE while those receiving endocrine therapy (HR 0.8; 95% CI, 0.8–0.8) had a lower risk of VTE when assessed as time‐varying covariates. Patients who identified as Black had a higher risk of VTE (HR 1.35; 95% CI, 1.32–1.38) compared to White patients, while patients who identified as Asian/Pacific Islander had a lower risk (HR 0.72; 95% CI, 0.68–0.76). Rurality, SVI, geographic region, marital status, primary language, renal function, and hepatic function did not meet the threshold for inclusion (Table S9).

### 3.4 Impact of CAT on Mortality

Patients in the overall cohort with stage I cancer had a five‐year survival rate of 89.8% while those with stage IV/metastatic cancer had a survival rate of 37.1%–46.2%. After adjusting for baseline confounders and systemic therapy, the time‐varying occurrence of PE (HR 2.4; 95% CI 2.4–2.5), LE‐DVT (HR 2.1; 95% CI 2.1–2.2), and UE‐DVT (HR 2.3; 95% CI 2.2–2.4) within the first year of cancer diagnosis were each independently associated with increased mortality within the same time window (Table 3). Cancer type and stage were also independently associated with increased mortality; pancreatic cancer (HR 7.62; 95% CI 7.33–7.93) and stage IV/metastatic (HR 6.48; 95% CI 6.24–6.74) had the highest risk.

**TABLE 3:** Multivariable Cox regression analysis for association between VTE onset and mortality.

Characteristic	Hazard ratio	95% confidence interval	*p*
VTE^a^			
No VTE	Ref	Ref	Ref
Pulmonary Embolism onset	2.44	2.38, 2.50	< 0.001
Lower Extremity DVT onset	2.11	2.05, 2.16	< 0.001
Upper Extremity DVT onset	2.30	2.22, 2.39	< 0.001
Age	1.36	1.35, 1.37	< 0.001
Sex			
Female	Ref	Ref	Ref
Male	1.2	1.19, 1.22	< 0.001
Underweight BMI	1.76	1.72, 1.80	< 0.001
Cancer type			
Breast	Ref	Ref	Ref
Pancreas	7.61	7.32, 7.91	< 0.001
Liver	6.80	6.52, 7.10	< 0.001
Central nervous system	5.88	5.55, 6.23	< 0.001
Upper gastrointestinal	5.56	5.34, 5.78	< 0.001
Bile and gallbladder	5.20	4.96, 5.45	< 0.001
Lung	4.12	3.98, 4.26	< 0.001
Sarcoma	3.58	3.38, 3.80	< 0.001
Bladder	3.10	2.98, 3.24	< 0.001
Cervix	3.09	2.89, 3.31	< 0.001
Head and neck	3.02	2.90, 3.15	< 0.001
Uterus	2.11	2.01, 2.21	< 0.001
Melanoma	1.80	1.70, 1.91	< 0.001
Acute myeloid leukemia	2.62	2.45, 2.82	< 0.001
Other cancer	2.25	2.16, 2.35	< 0.001
Lower gastrointestinal	1.75	1.69, 1.82	< 0.001
Chronic myeloid leukemia or myelodysplastic syndrome	1.73	1.63, 1.84	< 0.001
Acute lymphoblastic leukemia	1.54	1.36, 1.74	< 0.001
Ovary	1.53	1.44, 1.63	< 0.001
Aggressive lymphoma	1.46	1.38, 1.55	< 0.001
Kidney	1.39	1.32, 1.46	< 0.001
Testis	1.08	0.89, 1.29	0.4
Myeloma	1.04	0.98, 1.10	0.3
Indolent lymphoma	0.77	0.72, 0.83	< 0.001
Chronic lymphocytic leukemia	0.66	0.62, 0.71	< 0.001
Prostate	0.61	0.59, 0.64	< 0.001
Cancer stage	NA	NA	NA
Stage I	NA	NA	NA
Stage II	1.92	1.83, 2.01	< 0.001
Stage III	3.07	2.94, 3.20	< 0.001
Stage IV	6.33	6.10, 6.57	< 0.001
Unknown	2.22	2.14, 2.31	< 0.001
Unstageable	4.53	4.28, 4.79	< 0.001
Treatment^a^			
Chemotherapy	1.24	1.22, 1.26	< 0.001
Immune checkpoint inhibitor	1.52	1.50, 1.55	< 0.001
Targeted therapy	1.38	1.35, 1.40	< 0.001
Endocrine therapy	1.08	1.04, 1.12	< 0.001
History of Hospitalization	1.18	1.16, 1.19	< 0.001
NCI Comorbidity Index	1.49	1.48, 1.51	< 0.001
Labs^b^			
White blood cell count ≤ 11	Ref	Ref	Ref
White blood cell count > 11	1.27	1.25, 1.29	< 0.001
Hemoglobin ≥ 10	Ref	Ref	Ref
Hemoglobin < 10	1.39	1.37, 1.41	< 0.001
Platelet count ≥ 50	Ref	Ref	Ref
Platelet count < 50	1.82	1.75, 1.90	< 0.001
Total bilirubin ≤ 2.4	Ref	Ref	Ref
Total bilirubin > 2.4	1.27	1.23, 1.30	< 0.001
Albumin ≥ 3.5	Ref	Ref	Ref
Albumin < 3.5	1.59	1.57, 1.61	< 0.001
eGFR ≥ 30	Ref	Ref	Ref
eGFR < 30	1.49	1.45, 1.52	< 0.001

Abbreviations: DVT, deep vein thrombosis; eGFR, estimated glomerular filtration rate; Ref, reference; VTE, venous thromboembolism.

^a^ Time‐varying covariates within 1 year.

^b^ Missing lab values were modeled as a separate category and results are not shown.

## 4 Discussion

In this large, contemporary, nationwide cohort study, we provide the most comprehensive epidemiologic assessment of cancer‐associated VTE (CAT) in the US to date. Leveraging Epic Cosmos, which integrates both inpatient and outpatient EHR data from health systems across all 50 states from 2018 to 2024, we found that the 12‐month cumulative incidence of CAT was 3.7% but increased to 5.7% among those requiring early systemic therapy. The strongest risk factors were cancer type, advanced stage, and systemic therapy, particularly cytotoxic chemotherapy‐based regimens. Importantly, the occurrence of VTE within the first year of cancer diagnosis was independently associated with higher mortality, underscoring the clinical impact of thrombosis in this population.

Another notable finding was the increasing use of apixaban as a prescribed anticoagulant over time, accompanied by declining prescriptions of low molecular weight heparin, warfarin, and rivaroxaban. This is consistent with overall trends in VTE treatment in general [8] and in line with increasing data that apixaban is a safe and effective choice for CAT [9, 10]. Of note, the trends we observed were among the patients in our cohort who developed CAT and received a therapeutic anticoagulant prescription within 30 days. The remaining 21% without a prescription likely represent patients who had prolonged inpatient admission, died or transitioned to hospice within that timeframe, or those with bleeding events that contraindicated the use of anticoagulation.

Patients with pancreatic cancer, bile/gallbladder cancer, and ALL had the highest incidence of VTE at 12 months regardless of cancer treatment. The increased risk with ALL is likely related to treatment plans that typically require prolonged hospitalization and frequent use of central venous catheters, consistent with the finding that patients with ALL also had the highest incidence of UE‐DVT at 12 months. Cancer types with a more than two‐fold increase in VTE incidence among treated patients (bladder, sarcoma, testis, uterus, kidney, melanoma) may reflect the combined influence of advanced disease, which often necessitates systemic treatment, and the prothrombotic effects of the treatment itself. In our study, systemic treatments were broadly categorized as chemotherapy, immune checkpoint inhibitor, targeted therapy, and endocrine therapy. These categories are increasingly heterogenous however, and there may be important within‐group differences in their impact on VTE risk. Future studies that classify anti‐cancer treatments into more discrete categories could help clarify whether specific therapies are associated with differing levels of risk, thus helping to inform and personalize treatment selection for patients. Our analysis also confirmed known risk factors for VTE, including higher BMI, prior VTE, paralysis, and recent hospitalization. CBC abnormalities, a key component of VTE risk prediction scores, were shown to have an 8%–12% increased risk of VTE in this current study [11, 12]. Interestingly, low albumin was shown to have a 41% increased risk, likely reflecting the malnutrition or cachectic state of patients with advanced malignancy and thus serving as a representative biomarker for poor performance status [13].

SVI and rurality were not independently associated with VTE. The SVI incorporates measures of socioeconomic status, household characteristics, demographics, and housing type/transportation access to geographically map vulnerable communities in the US. Increased vulnerability (a higher SVI) has been associated with decreased cancer screening rates [14], increased cancer mortality [15], and greater risk of death from PE [16]. Similarly, rurality has been associated with disparities in VTE mortality and cancer outcomes, often attributed to factors such as older age, lower employment rates, and challenges accessing care in rural populations [17, 18]. In our cohort, most patients lived in HHS regions 4 and 5, which are the two most populous regions in the US [19], and 10% lived in rural areas (compared to 20% in the 2020 US census) [20]. Thus, while our cohort broadly reflects the socioeconomic and geographic distribution of the US population, it is not fully representative. Furthermore, SVI is a composite measure whose individual components overlap with other variables in our models, which may attenuate its independent effect. It is also possible that, after accounting for cancer type, stage, and comorbidities, socioeconomic factors exert less direct influence on VTE risk itself and instead contribute more indirectly through differences in disease presentation and overall health status. In general, both SVI and rurality are proxies for complex social determinants of health and their relationship to VTE risk in patients with cancer may not be fully captured in our dataset.

Several prior epidemiological studies reported rising rates of CAT over time, particularly in analyses spanning the late 1990s through the 2010s [4, 5]. In contrast, our study, which evaluated patients from 2018 to 2023, found that VTE incidence has remained stable in recent years. One possible explanation for these differences is the impact of evolving diagnostic practices. The introduction of ICD‐10 codes for upper‐extremity DVT in 2015, together with expansions of diagnostic coding and advances in cross‐sectional imaging that facilitated incidental detection of pulmonary emboli [21, 22], likely contributed to the appearance of increasing VTE incidence in earlier eras. By comparison, our contemporary analysis reflects a period when diagnostic codes and imaging advances were more stable and thus may provide a more reliable estimate of current CAT incidence in the US. The VA analysis, which evaluated patients between 2006 and 2021, also found no increase in the incidence of CAT over time [3].

It is also notable that the incidence of CAT has not decreased over time despite validated risk models for identifying which individuals are at increased risk [11, 12] and randomized controlled trials showing that anticoagulation prophylaxis can significantly reduce VTE incidence among at‐risk ambulatory patients with cancer [23, 24]. Risk‐directed prophylaxis is recommended by guidelines from the American Society of Clinical Oncology (ASCO) and the National Comprehensive Cancer Network (NCCN) [25, 26]. However, multiple studies show that thromboprophylaxis remains underutilized and often not targeted to those at highest risk [27, 28]. In our study, we excluded individuals already receiving anticoagulation at the time of cancer diagnosis. Given the timing, these patients were likely on treatment‐dose therapy rather than prophylaxis. This distinction cannot be fully verified in our data, however, and the exclusion of any patients receiving VTE prophylaxis could have modestly affected our findings. Ultimately, balancing the risk of bleeding against clotting in patients with cancer remains a challenge, though there are also opportunities to further refine prediction models and integrate EHR‐based risk tools into routine practice.

Our results extend prior epidemiologic studies of CAT, which have largely been limited by more homogeneous patient populations or geographic regions. In the US, prior studies relied on SEER‐Medicare data (restricted to older patients) [5], the Veterans Affairs (VA) database (predominantly male) [3], or state‐level registries such as the California Cancer Registry [29]. Outside the US, large registry‐based studies from Denmark, Sweden, and Canada have provided valuable insights but do not reflect the racial, ethnic, and socioeconomic diversity of the US population. Cosmos data is representative of the overall US census in terms of age, race, ethnicity, insurance coverage, and SVI. Moreover, many prior analyses relied primarily on inpatient data, limiting the capture of VTE events that occurred in the outpatient setting. By incorporating both inpatient and outpatient encounters, our study provides a more complete picture of CAT epidemiology in routine practice.

Our study has limitations. First, Cosmos only includes participating Epic health systems and outcomes may be missed if patients receive care elsewhere. We worked to mitigate this through strict inclusion criteria to ensure complete capture of longitudinal data. Reassuringly, our stage‐specific 5‐year survival rates were similar to those reported in SEER (Stage I: 89.8% vs. 90.0%; Stage IV/metastatic: 37.1%–46.2% vs. 33.0%, respectively), suggesting that missing data did not substantially bias our findings [30, 31]. With respect to systemic therapy ascertainment, although some patients did not have documented treatment within 90 days of diagnosis, early treatment rates for cancer types in which prompt therapy is routinely expected were comparable to population‐based SEER estimates, supporting reasonably complete capture of systemic therapy initiation in Cosmos. For example, 71.1% of patients with stage III colon cancer, 72.5% of those with stage III breast cancer, and 68.1% of patients with aggressive lymphoma received systemic treatment within 90 days, closely aligning with those reported by SEER [32]. Furthermore, the median continuous follow‐up time was 682 days for VTE and 1269 days for mortality, a sufficient timeframe for assessing short‐term outcomes such as VTE.

Second, as with other registry studies, outcome ascertainment relied on administrative codes and misclassification is possible. To mitigate this concern, we used a previously validated ICD‐10‐CM‐based VTE phenotyping algorithm applied across inpatient and outpatient encounters, with a reported positive predictive value of 95%, sensitivity of 81% and a c‐statistic of 0.90 in US health systems [7]. Additionally, our observed VTE incidence was consistent with prior reports from other large epidemiology studies in US and Europe, supporting the reliability of our approach [3, 4, 5, 7].

Third, cancer diagnosis dates derived from EHR encounter data may not perfectly reflect the true clinical date of cancer diagnosis, particularly in referral‐based health systems and for indolent malignancies such as low‐risk prostate cancer of indolent lymphomas. Prior work has also shown only moderate concordance between EHR‐derived and registry‐based cancer diagnoses, with variability by cancer site [33]. In this study, we applied several cohort exclusions to prioritize diagnostic specificity; however, this approach may still introduce left truncation by anchoring caner onset to the first qualifying encounter rather than the true diagnostic date. Such imprecision in diagnosis timing is likely nondifferential with respect to VTE outcomes and would be more likely to bias effect estimates toward the null rather than inflate observed associations.

Fourth, cancer stage derived from EHR data is subject to substantial missingness and potential misclassification compared with gold‐standard cancer registry data. In our cohort, approximately half lacked structured stage information; therefore, we supplemented reported stage with ICD‐10‐CM‐based metastatic disease codes. Reassuringly, among patients with known stage, observed stage distributions for several common cancers—including breast, colorectal, and uterine cancers—were broadly consistent with population‐based SEER estimates, supporting the face validity of our approach despite known limitations of EHR based staging [34].

Finally, Cosmos is a dynamic dataset, with new organizations continuously joining and contributing to data over time. Thus the cohort may not be fully representative of the US population. In particular, White patients were overrepresented, while Asian patients and individuals identifying as Hispanic ethnicity were underrepresented relative to national estimates. While our results therefore reflect a snapshot in time and may have limited generalizability for certain subgroups, they nonetheless demonstrate the feasibility of leveraging a nationwide EHR network for large‐scale epidemiologic research.

## 5 Conclusion

We present the largest US‐based evaluation of CAT to date, spanning over 1.6 million patients across all 50 states. In this nationwide cohort of individuals with newly diagnosed cancer, patients with pancreatic and upper GI cancers, advanced stage, prior history of VTE, and those receiving cytotoxic chemotherapy had the highest risk of VTE. These findings highlight persistent high‐risk subgroups and underscore the importance of refining targeted prophylactic strategies. More broadly, this work demonstrates the feasibility of using a nationwide EHR database to conduct a wide‐scale epidemiological study to provide timely, representative epidemiologic assessments of CAT.

## Author Contributions

Conception and design: Ang Li and Omid Jafari. Collection and assembly of data: Ang Li, Omid Jafari, Rock Bum Kim, Shengling Ma, and Jun Y. Jiang. Data analysis: Ang Li, Omid Jafari, and Justine Ryu. Data interpretation: Ang Li, Omid Jafari, Justine Ryu, and Barbara D. Lam. Manuscript writing: Ang Li, Justine Ryu, and Barbara D. Lam. Final approval of manuscript: All authors.

## Funding

AL, a CPRIT Scholar in Cancer Research, was supported by Cancer Prevention and Research Institute of Texas (RR190104), NIH NHLBI (K23 HL159271, R01 HL180402), American Society of Hematology (ASH) Scholar Award, and a Career Development Award from Conquer Cancer, the ASCO Foundation.

## Ethics Statement

The study was deemed non‐human subject research by the Institutional Review Board at Baylor College of Medicine.

## Consent

All data is derived from the Epic Cosmos database. Patient identifiers are removed before any data is moved from a healthcare organization. Data protection goes beyond current HIPAA requirements. No user can access Cosmos data without attesting to their use and disclosing funding sources. Users can access only fully deidentified data, and all actions in Cosmos are audited to ensure appropriate use.

## Conflicts of Interest

The authors declare no conflicts of interest.

## Supporting information


**Data S1:** Supporting Information.

## Data Availability

The data that support the findings of this study are openly available in Epic Cosmos at https://cosmos.epic.com/.
