# Supplementary material for: Epidemiology of Cancer‐Associated Venous Thromboembolism Across the United States
Source: Am J Hematol. 2026 Mar 8;101(5):1005–18. doi: 10.1002/ajh.70271 (PMC13055130; doi:10.1002/ajh.70271)
Supplement: Supplementary file 1 — Data S1: Supporting Information. [file AJH-101-1005-s001.docx]

**Supplemental Figure 1: Incidence of pulmonary embolism and lower-extremity deep vein thrombosis by cancer type**

**
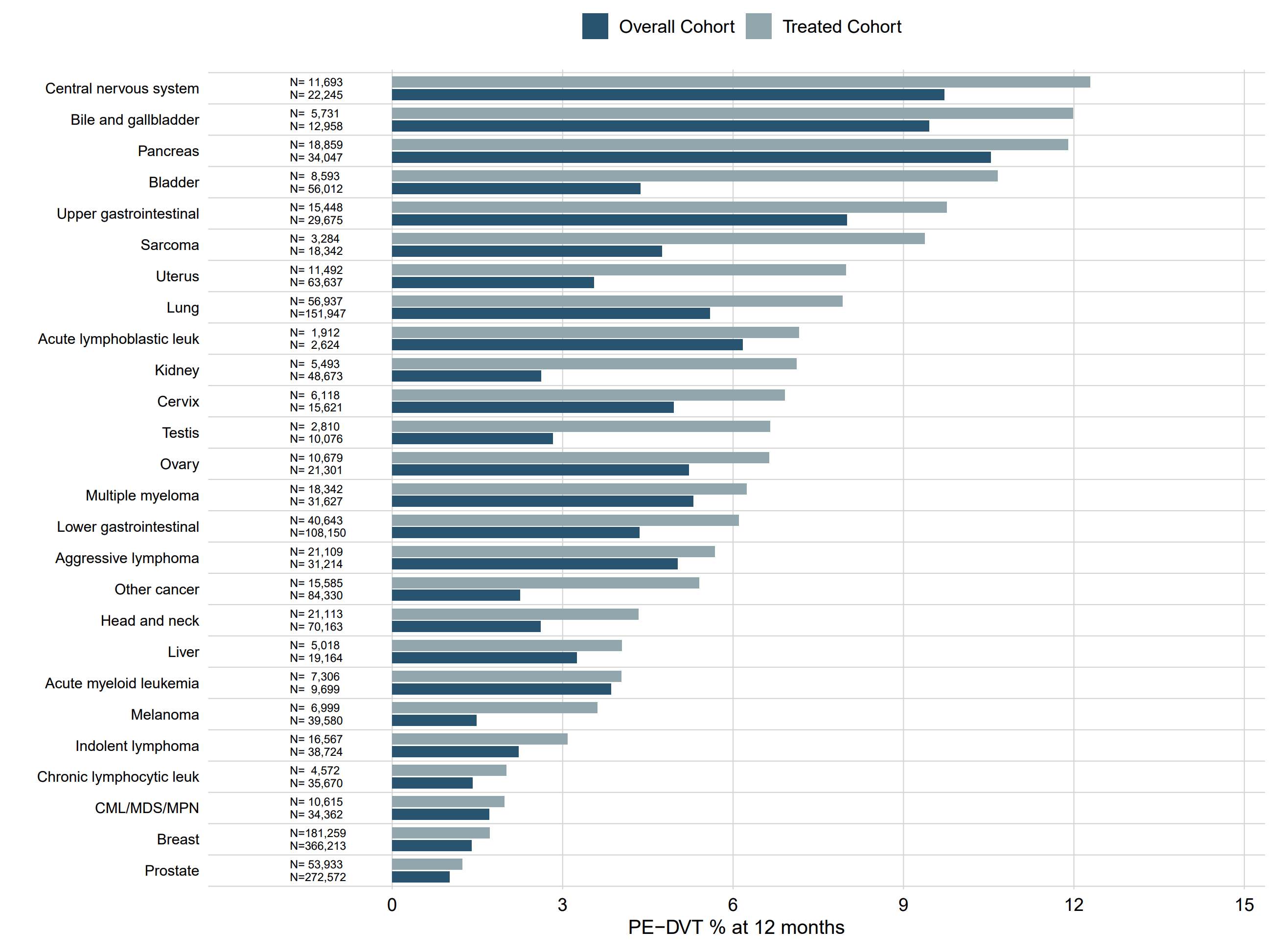
**

**Supplemental Figure 2: Incidence of upper-extremity deep vein thrombosis by cancer type**


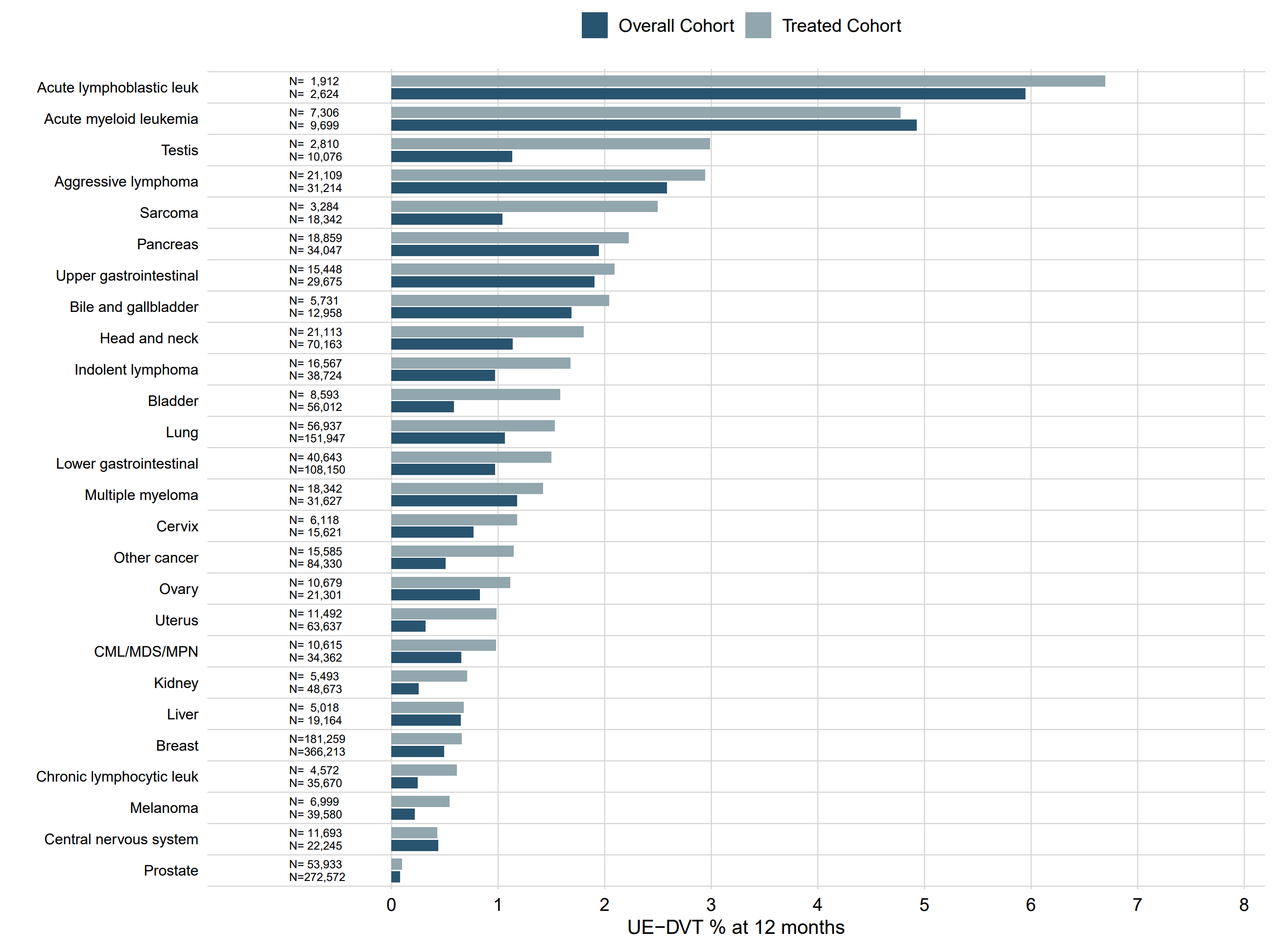


**Supplemental Table 1. Organization Filter**

| **Define high-quality sites:** |
| --- |
| Country = United States of America; “green lit” by Cosmos |
| **Define sites with at least 2 years of valid data and codes:** |
| Started data contribution before 1/1/2023 |
| Have ≥2 years of data with ≥1,000 monthly billing final diagnosis codes |
| **Define site with adequate inpatient & outpatient encounters in oncology departments:** |
| Have >1,000 inpatient encounters AND >1,000 emergency department visit encounters AND >1,000 outpatient face-to-face encounters AND >10,000 encounters at hematology or oncology departments |
| **Define non-pediatric sites:** |
| Average age of patients in the organization must be >22 years |

**Supplemental Table 2. Cohort Filters**

| **Define *International Classification of Diseases, Tenth Edition, Clinical Modification* (ICD-10-CM) codes for cancer** |
| --- |
| C__.%, C01, C07, C12, C19, C20, C23, C33, C37, C52, C55, C58, C61, C73, D46.%, D47.0%, D47.1%, D47.4%  Exclude: C44.%, C4A.%, C7B.1% (non-melanomatous skin cancer)  Between 1/1/2018 and 12/31/2023 |
| **Define eligible encounter type** |
| For billing final diagnosis:  Encounter type = ‘Hospital Encounter’ AND (IsHospitalAdmission = 1 OR IsEdVisit = 1 OR IsHospitalOutpatientVisit = 1) OR Encounter type IN (‘Diagnostic Services’, ‘Infusion’, ‘Procedure Visit’)  For encounter diagnosis:  Encounter type IN (‘Office Visit’, ‘Telemedicine’, ‘Anticoagulation Visit’, ‘Consult’, ‘Evaluation’, ‘Follow-Up’, ‘Procedural Consult’, ‘Surgical Consult’, ‘Transplant Evaluation’, ‘Transplant Follow Up’, ‘Urgent Care’, ‘Walk-In’, ‘Tumor Board Conference’) |
| **Define eligible department type** |
| IsHospitalAdmission = 1 (inpatient) OR DepartmentSpecialty LIKE ‘%Hematology%’, ‘%Oncology%’, ‘%Radiation%’, ‘%Infusion%’, ‘%Intensive%’, ‘%Critical%’, ‘%Surgery%’, ‘%Ear, Nose, Throat%’, ‘%Otolaryngology%’, ‘%Gynecology%’, ‘%Urology%’ (medical or surgical departments that diagnose and treat cancer)  Exclude: ‘%Neurology%’, ‘%Pediatric%’, ‘%Neonatal%’, ‘%Prenatal%’, ‘%Laboratory%’ |
| **Define cancer exclusion criteria** |
| Keep patients that have >=2 cancer diagnosis codes (excluding secondary/metastatic and unspecified) more than 30 days apart between 1/1/2018 and 12/31/2023 and >= 1 year from data contribution start date of organization  Exclude patients with polycythemia or essential thrombocythemia diagnosis  Exclude patients whose first cancer is not their most common cancer within 1 year of diagnosis  Exclude patients with any cancer diagnosis between 1/1/2017 and 12/31/2017 or within 1 year from data contribution start date of organization |
| **Define systemic therapy** |
| TherapeuticClass = ‘antineoplastics’ AND Mode = ‘inpatient’ or ‘outpatient’ (administered or prescribed)  Exclude: PharmaceuticalSubClass: ‘Dermatological%’ and ‘DMARD%’; SimpleGenericName: ‘BCG live%’, ‘sirolimus%’, ‘medroxyprogesterone%’, ‘megestrol%’, ‘iobenguane%’, ‘methoxsalen%’; Route: ‘intra-pyelocalyceal’, ‘intravesical’  Exclude: patients with inpatient, outpatient, or historical mode systemic therapies before cancer diagnosis date |
| **Define final cohort exclusion** |
| Exclude patients whose age at cancer diagnosis is <18 or >100  Exclude patients if not having at least 1 inpatient or outpatient “face-to-face” encounter in the 12 months window before their systemic therapy start date  Exclude patients if not having any inpatient or outpatient “face-to-face” follow-up encounters after their systemic therapy start date  Exclude patients if having a recorded death date before their systemic therapy start date  Exclude patients if having a known stage 0 cancer in the past 1 year before their systemic therapy start date  Exclude patients if having been diagnosed with acute VTE in the past 6 months before index date  Exclude patients if having been prescribed with active anticoagulant at the time of index date |

**Supplemental Table 3. Systemic Therapy Classifications**

| **Cytotoxic Chemotherapy:** |
| --- |
| altretamine, asparaginase, azacitidine, bendamustine, bleomycin, busulfan, cabazitaxel, calaspargase, capecitabine, carboplatin, carmustine, chlorambucil, cisplatin, cladribine, clofarabine, cyclophosphamide, cytarabine, dacarbazine, dactinomycin, daunorubicin, decitabine, docetaxel, doxorubicin, epirubicin, eribulin, etoposide, floxuridine, fludarabine, fluorouracil, gemcitabine, hydroxyurea, idarubicin, ifosfamide, irinotecan, ixabepilone, lomustine, lurbinectedin, mechlorethamine, melphalan, mercaptopurine, methotrexate, mitomycin, mitoxantrone, nelarabine, omacetaxine, oxaliplatin, paclitaxel, pegaspargase, pemetrexed, pentostatin, porfimer, pralatrexate, procarbazine, streptozocin, temozolomide, teniposide, thioguanine, thiotepa, topotecan, trabectedin, trifluridine, valrubicin, vinblastine, vincristine, vinorelbine |
| **Targeted Therapy** |
| abemaciclib, acalabrutinib, adagrasib, afatinib, aflibercept, alectinib, alemtuzumab, alpelisib, amivantamab, arsenic, asciminib, avapritinib, axitinib, belantamab, belinostat, belzutifan, bevacizumab, bexarotene, binimetinib, blinatumomab, bortezomib, bosutinib, brentuximab, brigatinib, cabozantinib, capivasertib, capmatinib, carfilzomib, ceritinib, cetuximab, cobimetinib, copanlisib, crizotinib, dabrafenib, dacomitinib, daratumumab, dasatinib, demcizumab, dostarlimab, duvelisib, elacestrant, elotuzumab, elranatamab, enasidenib, encorafenib, enfortumab, entrectinib, epcoritamab, erdafitinib, erlotinib, everolimus, fedratinib, fruquintinib, futibatinib, gefitinib, gemtuzumab, gilteritinib, glasdegib, ibrutinib, ibritumomab, idelalisib, imatinib, infigratinib, inotuzumab, isatuximab, ivosidenib, ixazomib, lapatinib, larotrectinib, lenalidomide, lenvatinib, loncastuximab, lorlatinib, margetuximab, midostaurin, mirvetuximab, mobocertinib, mogamulizumab, momelotinib, mosunetuzumab, necitumumab, neratinib, nilotinib, niraparib, nirogacestat, obinutuzumab, ofatumumab, olaparib, olaratumab, olutasidenib, osimertinib, pacritinib, palbociclib, pamrevlumab, panitumumab, panobinostat, pazopanib, pemigatinib, pertuzumab, pexidartinib, pirtobrutinib, polatuzumab, pomalidomide, ponatinib, pralsetinib, quizartinib, ramucirumab, regorafenib, ribociclib, ripretinib, rituximab, romidepsin, rucaparib, ruxolitinib, sacituzumab, selinexor, selpercatinib, selumetinib, siltuximab, sonidegib, sorafenib, sotorasib, sunitinib, tafasitamab, tagraxofusp, talazoparib, talquetamab, tazemetostat, tebentafusp, teclistamab, telotristat, temsirolimus, tepotinib, thalidomide, tisotumab, tivozanib, tositumomab, trametinib, trastuzumab, tremelimumab, tretinoin, tucatinib, umbralisib, upifitamab, vandetanib, veliparib, vemurafenib, venetoclax, vismodegib, vorinostat, zanubrutinib, glofitamab, lazertinib, repotrectinib, tarlatamab, vorasidenib |
| **Endocrine Therapy** |
| abarelix, abiraterone, anastrozole, apalutamide, bicalutamide, cyproterone, darolutamide, degarelix, enzalutamide, estramustine, exemestane, flutamide, fulvestrant, goserelin, histrelin, letrozole, leuprolide, mitotane, nilutamide, relugolix, tamoxifen, testolactone, toremifene, triptorelin |
| **Immune Checkpoint Inhibitor** |
| atezolizumab, avelumab, cemiplimab, durvalumab, ipilimumab, nivolumab, pembrolizumab, toripalimab |
| **Immune-CAR-T or Other: Excluded from current study** |
| axicabtagene, brexucabtagene, ciltacabtagene, idecabtagene, lisocabtagene, nadofaragene, talimogene, tisagenlecleucel  aldesleukin, interferon, peginterferon, ropeginterferon, sipuleucel, lifileucel |

**Supplemental Table 4. Outcome Filters**

| **Define ICD-10-CM codes for VTE:** |
| --- |
| **VTE:**  **Acute PE:** I26.02, I26.09, I26.92, I26.93, I26.94, I26.99  **Acute LE-DVT:** I80.10, I80.11, I80.12, I80.13, I80.201, I80.202, I80.203, I80.209, I80.211, I80.212, I80.213, I80.219, I80.221, I80.222, I80.223, I80.229, I80.231, I80.232, I80.233, I80.239, I80.241, I80.242, I80.243, I80.249, I80.251, I80.252, I80.253, I80.259, I80.291, I80.292, I80.293, I80.299, I82.220, I82.401, I82.402, I82.403, I82.409, I82.411, I82.412, I82.413, I82.419, I82.421, I82.422, I82.423, I82.429, I82.431, I82.432, I82.433, I82.439, I82.441, I82.442, I82.443, I82.449, I82.451, I82.452, I82.453, I82.459, I82.461, I82.462, I82.463, I82.469, I82.491, I82.492, I82.493, I82.499, I82.4Y1, I82.4Y2, I82.4Y3, I82.4Y9, I82.4Z1, I82.4Z2, I82.4Z3, I82.4Z9  **Acute UE-DVT:** I82.210, I82.290, I82.621, I82.622, I82.623, I82.629, I82.A11, I82.A12, I82.A13, I82.A19, I82.B11, I82.B12, I82.B13, I82.B19, I82.C11, I82.C12, I82.C13, I82.C19, I82.601, I82.602, I82.603, I82.609  **SPVT:** I82.0, I82.1, I82.3, I82.890, I82.90, I80.3, I80.8, I80.9, I63.6, I67.6 (not used in current study) |
| **Define eligible encounter type:** |
| For billing final diagnosis:  Encounter type = ‘Hospital Encounter’ AND (IsHospitalAdmission = 1 OR IsEdVisit = 1 OR IsHospitalOutpatientVisit = 1)  OR Encounter type IN (‘Diagnostic Services’, ‘Infusion’, ‘Procedure Visit’)  For encounter diagnosis:  Encounter type IN (‘Office Visit’, ‘Telemedicine’, ‘Anticoagulation Visit’, ‘Consult’, ‘Evaluation’, ‘Follow-Up’, ‘Procedural Consult’, ‘Surgical Consult’, ‘Transplant Evaluation’, ‘Transplant Follow Up’, ‘Urgent Care’, ‘Walk-In’, ‘Tumor Board Conference’) |
| **Define outcome criteria:** |
| **VTE:** first Acute PE, Acute LE-DVT, Acute UE-DVT after index date from  Any inpatient face-to-face encounter  2+ outpatient face-to-face encounters that are >30 and <365 days apart |

**Abbreviations:** ICD-10-CM, *International Classification of Diseases, Tenth Edition, Clinical Modification*; VTE, venous thromboembolism; PE, pulmonary embolism; LE-DVT, lower extremity deep vein thrombosis; UE-DVT, upper extremity deep vein thrombosis; SPVT, splanchnic vein thromboses; ATE, arterial thromboembolism; MI, myocardial infarction; iCVD, ischemic cerebrovascular disease; GI, gastrointestinal

**Supplemental Table 5. Baseline variable definitions**

| **Variable** | **Criteria** | **Lookback Window** |
| --- | --- | --- |
| Age |  | At diagnosis |
| Sex |  | At diagnosis |
| Race/Ethnicity |  | At diagnosis |
| Marital status |  | At diagnosis |
| Language |  | At diagnosis |
| RUCA |  | At diagnosis |
| State |  | At diagnosis |
| SVI ranking |  | At diagnosis |
| BMI | BMI is calculated using last known height and weight closest to cancer diagnosis | -12 months to +3 months from diagnosis |
| Cancer type | Condensed into 26 categories | At diagnosis |
| Cancer stage | Highest value reported from CancerStagingFact (derived from BEACON)  Further supplemented by ICD (C78.%, C79.%, C80.%, C7B.%) | -12 months to +3 months from diagnosis |
| Systemic therapy | See Supplemental Table 3  Mode: inpatient, outaptient  Time-varying covariate that represent time to first drug in each class | 12 months after diagnosis |
| Anticoagulant | TherapeuticClass = ‘Anticoagulants’  Mode: outpatient, historical  Exclude: ‘Anticoagulants – Citrate-based, Heparins’ | 12 months before diagnosis |
| Antiplatelet | TherapeuticClass = ‘Antiplatelet Drugs’  Mode: outpatient, historical | 12 months before diagnosis |
| Recent prolonged hospitalization | Admission >3 days ending before diagnosis (history)  Admission >3 days ending after diagnosis (current) | 3 months before diagnosis |
| History of VTE | **Acute PE:** I26.02, I26.09, I26.92, I26.93, I26.94, I26.99  **Acute LE-DVT:** I80.10, I80.11, I80.12, I80.13, I80.201, I80.202, I80.203, I80.209, I80.211, I80.212, I80.213, I80.219, I80.221, I80.222, I80.223, I80.229, I80.231, I80.232, I80.233, I80.239, I80.241, I80.242, I80.243, I80.249, I80.251, I80.252, I80.253, I80.259, I80.291, I80.292, I80.293, I80.299, I82.220, I82.401, I82.402, I82.403, I82.409, I82.411, I82.412, I82.413, I82.419, I82.421, I82.422, I82.423, I82.429, I82.431, I82.432, I82.433, I82.439, I82.441, I82.442, I82.443, I82.449, I82.451, I82.452, I82.453, I82.459, I82.461, I82.462, I82.463, I82.469, I82.491, I82.492, I82.493, I82.499, I82.4Y1, I82.4Y2, I82.4Y3, I82.4Y9, I82.4Z1, I82.4Z2, I82.4Z3, I82.4Z9  **Acute UE-DVT:** I82.210, I82.290, I82.621, I82.622, I82.623, I82.629, I82.A11, I82.A12, I82.A13, I82.A19, I82.B11, I82.B12, I82.B13, I82.B19, I82.C11, I82.C12, I82.C13, I82.C19, I82.601, I82.602, I82.603, I82.609  **Acute Other VTE:** I82.0, I82.1, I82.3, I82.890, I82.90, I80.3, I80.8, I80.9, I63.6, I67.6  **Historic VTE:** Z86.711, Z86.718  **Chronic PE:** I27.82  **Chronic DVT:** I82.211, I82.221, I82.291, I82.501, I82.502, I82.503, I82.509, I82.511, I82.512, I82.513, I82.519, I82.521, I82.522, I82.523, I82.529, I82.531, I82.532, I82.533, I82.539, I82.541, I82.542, I82.543, I82.549, I82.551, I82.552, I82.553, I82.559, I82.561, I82.562, I82.563, I82.569, I82.591, I82.592, I82.593, I82.599, I82.5Y1, I82.5Y2, I82.5Y3, I82.5Y9, I82.5Z1, I82.5Z2, I82.5Z3, I82.5Z9, I82.701, I82.702, I82.703, I82.709, I82.721, I82.722, I82.723, I82.729, I82.891, I82.91, I82.A21, I82.A22, I82.A23, I82.A29, I82.B21, I82.B22, I82.B23, I82.B29, I82.C21, I82.C22, I82.C23, I82.C29  **# Exclusion criteria:** acute PE, acute LE-DVT, acute UE-DVT, or acute other VTE <= 6 months before diagnosis  **# Recent history of VTE:** acute PE, acute LE-DVT, acute UE-DVT, or acute other VTE 6-12 months before diagnosis  **# Remote history of VTE:** acute PE, acute LE-DVT, acute UE-DVT, or acute other VTE >12 months before diagnosis OR chronic PE, chronic DVT, or historic VTE anytime before diagnosis – this is treated as History of VTE | Lifetime before diagnosis |
| History of bleeding | **Intracranial hemorrhage:** I60.00, I60.01, I60.02, I60.10, I60.11, I60.12, I60.2, I60.20, I60.21, I60.22, I60.30, I60.31, I60.32, I60.4, I60.50, I60.51, I60.52, I60.6, I60.7, I60.8, I60.9, I61.0, I61.1, I61.2, I61.3, I61.4, I61.5, I61.6, I61.8, I61.9, I62.00, I62.01, I62.02, I62.03, I62.1, I62.9, S06.340A, S06.341A, S06.342A, S06.343A, S06.344A, S06.345A, S06.346A, S06.347A, S06.348A, S06.349A, S06.350A, S06.351A, S06.352A, S06.353A, S06.354A, S06.355A, S06.356A, S06.357A, S06.358A, S06.359A, S06.360A, S06.361A, S06.362A, S06.363A, S06.364A, S06.365A, S06.366A, S06.367A, S06.368A, S06.369A, S06.370A, S06.371A, S06.372A, S06.373A, S06.374A, S06.375A, S06.376A, S06.377A, S06.378A, S06.379A, S06.380A, S06.381A, S06.382A, S06.383A, S06.384A, S06.385A, S06.386A, S06.387A, S06.388A, S06.389A, S06.4X0A, S06.4X1A, S06.4X2A, S06.4X3A, S06.4X4A, S06.4X5A, S06.4X6A, S06.4X7A, S06.4X8A, S06.4X9A, S06.4XAA, S06.5X0A, S06.5X1A, S06.5X2A, S06.5X3A, S06.5X4A, S06.5X5A, S06.5X6A, S06.5X7A, S06.5X8A, S06.5X9A, S06.6X0A, S06.6X1A, S06.6X2A, S06.6X3A, S06.6X4A, S06.6X5A, S06.6X6A, S06.6X7A, S06.6X8A, S06.6X9A  **Intraarticular:** M25.00, M25.011, M25.012, M25.019, M25.021, M25.022, M25.029, M25.031, M25.032, M25.039, M25.041, M25.042, M25.049, M25.051, M25.052, M25.059, M25.061, M25.062, M25.069, M25.071, M25.072, M25.073, M25.074, M25.075, M25.076, M25.08  **Intraocular:** H05.231, H05.232, H05.233, H05.239, H21.0, H31.301, H31.302, H31.303, H31.309, H31.311, H31.312, H31.313, H31.319, H31.411, H31.412, H31.413, H31.419, H35.60, H35.61, H35.62, H35.63, H35.731, H35.732, H35.733, H35.739, H43.10, H43.11, H43.12, H43.13, H44.81, H47.021, H47.022, H47.023, H47.029  **Lower GI:** K50.011, K50.111, K50.811, K50.911, K51.011, K51.211, K51.311, K51.411, K51.511, K51.811, K51.911, K55.21, K57.01, K57.11, K57.13, K57.21, K57.31, K57.33, K57.41, K57.51, K57.53, K57.81, K57.91, K57.93, K62.5, K63.81, K92.1, K92.2, K94.01, K94.11  **Upper GI:** I85.01, I85.11, K22.11, K22.6, K25.0, K25.2, K25.4, K25.6, K26.0, K26.2, K26.4, K26.6, K27.0, K27.2, K27.4, K27.6, K28.0, K28.2, K28.4, K28.6, K29.01, K29.21, K29.31, K29.41, K29.51, K29.61, K29.71, K29.81, K29.91, K31.811, K31.82, K92.0, K94.21, K94.31  **Pericardial:** I23.0, I31.2, S26.00XA, S26.01XA, S26.020A, S26.021A, S26.022A, S26.09XA  **Respiratory:** J95.01, R04.1, R04.2, R04.89, R04.9  **Retroperitoneal:** K66.1, K68.3  **Soft Tissue:** M79.81  **Thoracic:** J94.2, S27.1XXA  **Ear:** H61.121, H61.122, H61.123, H61.129, H92.20, H92.21, H92.22, H92.23  **Genitourinary:** N30.41, N42.1, N99.510, N99.520, N99.530, R31.0  **Gynecologic:** O71.7, N83.6, N83.7, N93.8, N93.9  **Miscellaneous:** R58  **# Recent history of bleed:** within 12 months before diagnosis  **# Remote history of bleed:** >12 months before diagnosis | Lifetime before diagnosis |
| CBC:   1. WBC 2. Hgb 3. Plt | LOINC:   1. 804-5, 6690-2, 12227-5, 26464-8, 33256-9, 46498-9 2. 718-7, 14775-1, 20509-6, 30313-1, 30350-3, 30351-1, 30352-9, 55782-7, 76768-1, 76769-9, 97556-5, 97550-8, 97555-7 3. 777-3, 778-1, 13056-7, 26515-7, 26516-5, 49497-1, 74775-8, 74464-9, 97995-5 | -3 months to +1 month from diagnosis |
| CMP:   1. Cr 2. Alb 3. ALT 4. TB | LOINC:   1. 2160-0, 21232-4, 38483-4 2. 1751-7, 2862-1, 61151-7, 61152-5, 76631-1, 77148-5, 101198-0, 103577-3 3. 1742-6, 1743-4, 1744-2, 76625-3, 77144-4 4. 1975-2, 42719-5, 59827-6, 59828-4 | -3 months to +1 month from diagnosis |
| NCI Comorbidity Index (NCI-CI) | AcuteMi: I21.%, I22.%  HistoryMi: I25.2  CHF: I09.9, I11.0, I13.0, I13.2, I25.5, I42.0, I43, P29.0, I42.[5-9], I50%  PVD: I7[0-1]%, I73.[189]%, 77.1, I79.[02], K55.[189], Z95.[8-9]%  CVD: G4[56]%, H34.0%, I6%  COPD: I27.[89]%, J[46][0-7]%, J68.4, J70.[13]  Dementia: F0[0-3]%, F05.1, G30%, G31.1  Paralysis: G04.1, G11.4, G80.[12], G8[12]%, G83.[0-49]%  Diabetes: E1[013].[01689]%  DiabetesComp: E1[013].[2-57]%  RenalDisease: I12.0, I13.1%, N0[35].[2-7], N1[8-9]%, N25.0, Z49.[0-2], Z94.0, Z99.2  MildLiverDisease: B18%, K70.[0-39]%, K71.[3-57]%, K7[34]%, K76.[02-4]%, Z94.4  LiverDisease: I85.[09]%, I86.4, I98.2, K70.4%, K71.1%, K72.[19]%, K76.[5-7]  Ulcers: K2[5-8]%  RheumDisease: M0[56]%, M31.5, M3[2-4]%, M35.[13]%, M36.0  HIVAIDS: B2[0-24]  NCI_CI = AcuteMi * 0.12624 + HistoryMi * 0.07999 + CHF * 0.64441 + PVD * 0.26232 + CVD * 0.27868 + COPD * 0.52487 + Dementia * 0.72219 + Paralysis * 0.39882 + GREATEST(Diabetes, DiabetesComp) * 0.29408 + RenalDisease * 0.47010 + GREATEST(MildLiverDisease, LiverDisease) * 0.73803 + Ulcers * 0.07506 + RheumDisease * 0.21905 + HIVAIDS * 0.58362 | 12 months before diagnosis |

**Abbreviations:** RUCA, Rural-Urban Commuting Area; SVI, Social Vulnerability Index; d, days; VTE, venous thromboembolism; PE, pulmonary embolism; DVT, deep vein thrombosis; LE-DVT, lower extremity deep vein thrombosis; UE-DVT, upper extremity deep vein thrombosis; CBC, complete blood count; WBC, white blood cell; Hgb, hemoglobin; Plt, platelets; LOINC, Logical Observation Identifiers, Names, and Codes; CMP, complete metabolic panel; Cr, creatinine; Alb, albumin; ALT, alanine transaminase; TB, total bilirubin

**Supplemental Table 6: Additional baseline patient characteristics**

| **Characteristic** | **Overall**  **N = 1,628,626** | **Treated**  **N = 562,110** |
| --- | --- | --- |
| **Primary Language** | | |
| English | 1,552,764 (95.3%) | 532,776 (94.8%) |
| Other than English | 75,862 (4.7%) | 29,334 (5.2%) |
| **Marital Status** | | |
| With partner | 957,521 (58.8%) | 326,031 (58.0%) |
| No partner | 649,554 (39.9%) | 229,865 (40.9%) |
| Unknown | 21,551 (1.3%) | 6,214 (1.1%) |
| **Urban Rural Class (by RUCA code)** | | |
| Metropolitan | 1,308,212 (80.3%) | 449,799 (80.0%) |
| Micropolitan | 164,491 (10.1%) | 57,096 (10.2%) |
| Rural | 148,733 (9.1%) | 53,811 (9.6%) |
| Unknown | 7,190 (0.4%) | 1,404 (0.2%) |
| **Comorbidities/Medical History** | | |
| Myocardial Infarction | 67,666 (4.2%) | 21,073 (3.7%) |
| Cerebrovascular Disease | 93,552 (5.7%) | 29,342 (5.2%) |
| Peripheral Vascular Disease | 136,020 (8.4%) | 42,444 (7.6%) |
| Congestive Heart Failure | 103,006 (6.3%) | 31,127 (5.5%) |
| Diabetes | 319,766 (19.6%) | 105,958 (18.9%) |
| Paralysis | 12,186 (0.7%) | 4,725 (0.8%) |
| Renal Disease | 162,181 (10.0%) | 49,090 (8.7%) |
| Liver Disease | 86,156 (5.3%) | 28,501 (5.1%) |
| Ulcers | 22,903 (1.4%) | 8,680 (1.5%) |
| Rheumatologic Disease | 37,610 (2.3%) | 12,940 (2.3%) |
| HIV or AIDS | 5,243 (0.3%) | 2,224 (0.4%) |
| COPD | 294,232 (18.1%) | 99,918 (17.8%) |
| **Baseline Anticoagulation** | | |
| Antiplatelet use | 131,806 (8.1%) | 42,910 (7.6%) |
| CYP3A4 use | 27,248 (1.7%) | 8,724 (1.6%) |
| **Hospitalization** | | |
| Hospitalization (>3 days) within last 90 days | 186,366 (11.4%) | 77,268 (13.7%) |
| Current hospitalization | 119,831 (7.4%) | 44,812 (8.0%) |
| Prior hospitalization | 94,548 (5.8%) | 41,139 (7.3%) |
| **Additional VTE and Bleeding History** | | |
| VTE history more than 1 year prior | 42,031 (2.6%) | 12,868 (2.3%) |
| Bleeding history more than 1 year prior | 69,361 (4.3%) | 22,387 (4.0%) |
| **Labs** | | |
| White blood cell count median (Q1, Q3) | 7.5 (5.9, 9.8) | 7.5 (5.9, 9.7) |
| White blood cell count ≤ 11 | 926,055 (56.9%) | 380,510 (67.7%) |
| White blood cell count missing | 508,232 (31.2%) | 107,294 (19.1%) |
| Hemoglobin median (Q1, Q3) | 13.1 (11.6, 14.2) | 13.0 (11.5, 14.1) |
| Hemoglobin ≥ 10 | 1,011,279 (62.1%) | 404,685 (72.0%) |
| Hemoglobin missing | 492,033 (30.2%) | 102,266 (18.2%) |
| Platelet count median (Q1, Q3) | 247.0 (198.0, 306.0) | 259.0 (206.0, 321.0) |
| Platelet count ≥ 350 | 161,415 (9.9%) | 83,067 (14.8%) |
| Platelet count missing | 487,061 (29.9%) | 99,618 (17.7%) |
| Alanine aminotransferase median (Q1, Q3) | 20,0 (14,0, 29.0) | 20.0 (14.0, 29.0) |
| Alanine aminotransferase ≤ 260 | 973,834 (59.8%) | 424,540 (75.5%) |
| Alanine aminotransferase missing | 647,449 (39.8%) | 133,933 (23.8%) |
| Total bilirubin median (Q1, Q3) | 0.5 (0.4, 0.7) | 0.5 (0.3, 0.7) |
| Total bilirubin ≤ 2.4 | 929,149 (57.1%) | 407,928 (72.6%) |
| Total bilirubin missing | 681,045 (41.8%) | 145,767 (25.9%) |
| Albumin median (Q1, Q3) | 4.0 (3.6, 4.3) | 4.0 (3.6, 4.3) |
| Albumin ≥ 3.5 | 805,895 (49.5%) | 345,938 (61.5%) |
| Albumin missing | 652,475 (40.1%) | 138,871 (24.7%) |
| Creatinine median (Q1, Q3) | 0.9 (0.7, 1.1) | 0.8 (0.7, 1.0) |
| Creatinine ≤ 2 | 1,145.8987 (70.4%) | 458,279 (81.5%) |
| Creatining missing | 448,778 (27.6%) | 92,571 (16.5%) |
| eGFR ≥ 30 | 1,139,198 (69.9%) | 457,643 (81.4%) |
| eGFR missing | 453,883 (27.9%) | 93,138 (16.6%) |

**Abbreviations**: RUCA, rural-urban commuting area codes; HIV, human immunodeficiency virus; AIDS, acquired immunodeficiency syndrome; COPD, chronic obstructive pulmonary disease; VTE, venous thromboembolism; BMI, body mass index; eGFR, estimated glomerular filtration rate; LDL, low-density lipoprotein

**Supplemental Table 7: Cumulative incidence of VTE at 12 months by cancer type**

(A) Among all patients (n=1,628,626)

| **Cancer type** | **N** | **CI VTE** | **CI PE/LE-DVT** | **CI UE-DVT** |
| --- | --- | --- | --- | --- |
| Pancreas | 34,047 | 12.0% | 10.5% | 1.9% |
| ALL | 2,624 | 11.1% | 6.2% | 5.9% |
| Bile gallbladder | 12,958 | 10.8% | 9.5% | 1.7% |
| CNS | 22,245 | 10.0% | 9.7% | 0.4% |
| Upper GI | 29.675 | 9.5% | 8.0% | 1.9% |
| AML | 9,699 | 8.4% | 3.9% | 4.9% |
| Aggressive lymphoma | 31,214 | 7.3% | 5.0% | 2.6% |
| Lung | 151,947 | 6.5% | 5.6% | 1.1% |
| Myeloma | 31,627 | 6.3% | 5.3% | 1.2% |
| Ovary | 21,301 | 5.9% | 5.2% | 0.8% |
| Cervix | 15,621 | 5.6% | 5.0% | 0.8% |
| Sarcoma | 18,342 | 5.5% | 4.8% | 1.0% |
| Lower GI | 108,150 | 5.1% | 4.4% | 1.0% |
| Bladder | 56,012 | 4.8% | 4.4% | 0.6% |
| Uterus | 63,637 | 3.8% | 3.6% | 0.3% |
| Liver | 19,164 | 3.8% | 3.3% | 0.7% |
| Testis | 10,076 | 3.8% | 2.8% | 1.1% |
| Head neck | 70,163 | 3.6% | 2.6% | 1.1% |
| Indolent lymphoma | 38,724 | 3.1% | 2.2% | 1.0% |
| Kidney | 48,673 | 2.8% | 2.6% | 0.3% |
| Other cancer | 84,330 | 2.7% | 2.3% | 0.5% |
| CML/MDS/MPN | 34,362 | 2.2% | 1.7% | 0.7% |
| Breast | 366,213 | 1.8% | 1.4% | 0.5% |
| Melanoma | 39,580 | 1.7% | 1.5% | 0.2% |
| CLL | 35,670 | 1.6% | 1.4% | 0.2% |
| Prostate | 272,572 | 1.1% | 1.0% | 0.1% |

(B) Among patients who received systemic treatment (n=562,110)

| **Cancer type** | **N** | **CI VTE** | **CI PE/LE-DVT** | **CI UE-DVT** |
| --- | --- | --- | --- | --- |
| Pancreas | 34,047 | 13.6% | 11.9% | 2.2% |
| Bile gallbladder | 12,958 | 13.6% | 12.0% | 2.0% |
| ALL | 2,624 | 12.7% | 7.2% | 6.7% |
| CNS | 22,245 | 12.5% | 12.3% | 0.4% |
| Bladder | 56,012 | 11.9% | 10.7% | 1.6% |
| Sarcoma | 18,342 | 11.4% | 9.4% | 2.5% |
| Upper GI | 29,675 | 11.4% | 9.8% | 2.1% |
| Lung | 151,947 | 9.2% | 7.9% | 1.5% |
| Testis | 10,076 | 9.2% | 6.7% | 3.0% |
| Uterus | 63,637 | 8.8% | 8.0% | 1.0% |
| AML | 9,699 | 8.4% | 4.0% | 4.8% |
| Aggressive lymphoma | 31,214 | 8.2% | 5.7% | 2.9% |
| Cervix | 15,621 | 7.9% | 6.9% | 1.2% |
| Kidney | 48,673 | 7.6% | 7.1% | 0.7% |
| Myeloma | 31,627 | 7.5% | 6.2% | 1.4% |
| Ovary | 21,301 | 7.5% | 6.6% | 1.1% |
| Lower GI | 108,150 | 7.3% | 6.1% | 1.5% |
| Other cancer | 84,330 | 6.4% | 5.4% | 1.1% |
| Head neck | 70,163 | 5.9% | 4.3% | 1.8% |
| Liver | 19,164 | 4.6% | 4.0% | 0.7% |
| Indolent lymphoma | 38,724 | 4.6% | 3.1% | 1.7% |
| Melanoma | 39,580 | 4.0% | 3.6% | 0.5% |
| CML/MDS/MPN | 34,362 | 2.8% | 2.0% | 1.0% |
| CLL | 35,670 | 2.5% | 2.0% | 0.6% |
| Breast | 366,213 | 2.3% | 1.7% | 0.7% |
| Prostate | 272,572 | 1.3% | 1.2% | 0.1% |

**Supplemental Table 8: Anticoagulant prescription use over time**

| **Year** | **Apixaban** | **Rivaroxaban** | **Edoxaban/ dabigatran** | **Low molecular weight heparin** | **Warfarin** | **Unknown** |
| --- | --- | --- | --- | --- | --- | --- |
| 2018 | 24.2% | 17.7% | 0.654% | 29.6% | 6.48% | 21.4% |
| 2019 | 35.8% | 15.7% | 0.337% | 23.2% | 4.21% | 20.7% |
| 2020 | 45.2% | 14.4% | 0.212% | 16.5% | 2.35% | 21.3% |
| 2021 | 52.3% | 12.5% | 0.088% | 11.8% | 1.65% | 21.7% |
| 2022 | 57.3% | 10.5% | 0.073% | 9.85% | 1.23% | 21.1% |
| 2023 | 62.1% | 7.5% | 0.074% | 8.51% | 0.87% | 20.9% |

**Supplemental Table 9: Unadjusted univariable analysis of association with cancer-associated thrombosis**

| **Variable** | **Hazard Ratio** | **95% Confidence Interval** | **p-value** |
| --- | --- | --- | --- |
| **Age** | 1.03 | 1.03 - 1.04 | <0.00001 |
| **Sex** | | | |
| Male | 1.06 | 1.05 - 1.08 | <0.00001 |
| **Race** | | | |
| Black | 1.37 | 1.34 - 1.40 | <0.00001 |
| Asian Pacific Islander | 0.67 | 0.64 - 0.71 | <0.00001 |
| American Indian/ Alaska Native | 0.99 | 0.91 - 1.09 | 0.8748 |
| Other | 1.00 | 0.94 - 1.06 | 0.9115 |
| Unknown | 0.80 | 0.72 - 0.89 | <0.00001 |
| **Ethnicity** | | | |
| Hispanic | 0.98 | 0.95 - 1.02 | 0.2761 |
| Unknown | 0.89 | 0.84 - 0.93 | <0.00001 |
| **Marital Status** | | | |
| No partner | 1.16 | 1.14 - 1.18 | <0.00001 |
| Unknown | 0.96 | 0.88 - 1.03 | 0.2475 |
| **Primary Language** | | | |
| Non-English | 1.02 | 0.98 - 1.06 | 0.257 |
| **Urban Rural Class (by RUCA code)** | | | |
| Micropolitan | 0.96 | 0.94 - 0.99 | 0.0046 |
| Rural | 0.95 | 0.92 - 0.98 | 0.0005 |
| Unknown | 0.87 | 0.76 - 0.99 | 0.0348 |
| **State Region (HHS Classification)*** | | | |
| Region 2 | 1.04 | 1.00 - 1.09 | 0.0724 |
| Region 3 | 1.16 | 1.12 - 1.20 | <0.00001 |
| Region 4 | 1.08 | 1.04 - 1.12 | 0.0001 |
| Region 5 | 1.24 | 1.20 - 1.29 | <0.00001 |
| Region 6 | 1.23 | 1.18 - 1.28 | <0.00001 |
| Region 7 | 1.12 | 1.07 - 1.17 | <0.00001 |
| Region 8 | 1.23 | 1.17 - 1.29 | <0.00001 |
| Region 9 | 0.96 | 0.91 - 1.00 | 0.0779 |
| Region 10 | 1.02 | 0.96 - 1.08 | 0.5337 |
| Unknown | 1.00 | 0.45 - 2.22 | 0.9948 |
| **Social Vulnerability Index Quartile** | | | |
| Mid-Low | 1.02 | 1.00 - 1.04 | 0.1079 |
| Mid-High | 1.07 | 1.05 - 1.10 | <0.00001 |
| High | 1.19 | 1.17 - 1.22 | <0.00001 |
| Missing | 0.98 | 0.89 - 1.09 | 0.7695 |
| **Cancer Type** | | | |
| Prostate | 0.60 | 0.58 - 0.63 | <0.00001 |
| Head and neck | 2.06 | 1.97 - 2.16 | <0.00001 |
| Lung | 3.90 | 3.78 - 4.02 | <0.00001 |
| Lower gastrointestinal | 2.98 | 2.88 - 3.09 | <0.00001 |
| Upper gastrointestinal | 6.04 | 5.78 - 6.31 | <0.00001 |
| Pancreas | 8.00 | 7.70 - 8.32 | <0.00001 |
| Liver | 2.43 | 2.25 - 2.63 | <0.00001 |
| Bile and gallbladder | 7.13 | 6.73 - 7.55 | <0.00001 |
| Kidney | 1.64 | 1.55 - 1.74 | <0.00001 |
| Bladder | 2.80 | 2.68 - 2.93 | <0.00001 |
| Testis | 2.20 | 1.98 - 2.43 | <0.00001 |
| Cervix | 3.28 | 3.06 - 3.52 | <0.00001 |
| Ovary | 3.36 | 3.17 - 3.57 | <0.00001 |
| Uterus | 2.19 | 2.09 - 2.29 | <0.00001 |
| Central nervous system | 6.34 | 6.04 - 6.65 | <0.00001 |
| Melanoma | 0.97 | 0.90 - 1.06 | 0.5217 |
| Sarcoma | 3.28 | 3.07 - 3.51 | <0.00001 |
| Myeloma | 3.65 | 3.47 - 3.84 | <0.00001 |
| Acute myeloid leukemia | 5.36 | 4.99 - 5.77 | <0.00001 |
| Acute lymphoblastic leukemia | 6.66 | 5.92 - 7.49 | <0.00001 |
| Chronic myeloid leukemia or myelodysplastic syndrome | 1.29 | 1.20 - 1.39 | <0.00001 |
| Chronic lymphocytic leukemia | 0.90 | 0.83 - 0.98 | 0.0149 |
| Aggressive lymphoma | 4.25 | 4.06 - 4.46 | <0.00001 |
| Indolent lymphoma | 1.71 | 1.61 - 1.82 | <0.00001 |
| Other cancer | 1.54 | 1.47 - 1.62 | <0.00001 |
| **Cancer Stage** | | | |
| Stage I | 2.22 | 2.11 - 2.33 | <0.00001 |
| Stage II | 3.96 | 3.78 - 4.14 | <0.00001 |
| Stage III | 6.82 | 6.52 - 7.12 | <0.00001 |
| Stage IV | 6.61 | 6.35 - 6.88 | <0.00001 |
| Metastatic ICD | 3.20 | 3.07 - 3.33 | <0.00001 |
| Unstageable | 1.63 | 1.57 - 1.69 | <0.00001 |
| With metastatic brain lesions | 4.36 | 4.19 - 4.55 | <0.00001 |
| **Concurrent Anticoagulation** | | | |
| Antiplatelet | 1.16 | 1.13 - 1.19 | <0.00001 |
| CYP3A4 Inhibitors | 0.94 | 0.88 - 1.00 | 0.0567 |
| **Hospitalization** | | | |
| Has Hospitalization | 2.74 | 2.69 - 2.79 | <0.00001 |
| Current Hospitalization | 2.52 | 2.47 - 2.58 | <0.00001 |
| History of Hospitalization | 2.58 | 2.52 - 2.64 | <0.00001 |
| **History of Bleeding or Clotting** | | | |
| VTE History | 2.62 | 2.54 - 2.70 | <0.00001 |
| ATE History | 1.23 | 1.19 - 1.27 | <0.00001 |
| Bleeding History | 1.52 | 1.49 - 1.55 | <0.00001 |
| **NCI Comorbidity Index** | | | |
| 0 | 1.42 | 1.40 - 1.44 | <0.00001 |
| >0-0.5 | 1.38 | 1.35 - 1.42 | <0.00001 |
| >0.5-1 | 1.54 | 1.51 - 1.57 | <0.00001 |
| >1 | 1.72 | 1.68 - 1.76 | <0.00001 |
| **Comorbidities** | | | |
| Myocardial Infarction | 1.38 | 1.33 - 1.43 | <0.00001 |
| Cerebrovascular Disease | 1.27 | 1.23 - 1.31 | <0.00001 |
| Peripheral Vascular Disease | 1.30 | 1.27 - 1.33 | <0.00001 |
| Congestive Heart Failure | 1.44 | 1.40 - 1.49 | <0.00001 |
| Diabetes | 1.26 | 1.24 - 1.29 | <0.00001 |
| Paralysis | 2.61 | 2.45 - 2.77 | <0.00001 |
| Renal Disease | 1.35 | 1.32 - 1.39 | <0.00001 |
| Liver Disease | 1.27 | 1.23 - 1.31 | <0.00001 |
| Ulcers | 1.77 | 1.68 - 1.87 | <0.00001 |
| Rheumatologic Disease | 1.29 | 1.23 - 1.35 | <0.00001 |
| HIV or AIDS | 1.45 | 1.29 - 1.63 | <0.00001 |
| COPD | 1.42 | 1.39 - 1.45 | <0.00001 |
| **Body Mass Index** | | | |
| Normal BMI | 1.01 | 1.01 - 1.01 | <0.00001 |
| Underweight | 1.29 | 1.22 - 1.36 | <0.00001 |
| Overweight (BMI 25-30) | 0.97 | 0.95 - 0.99 | 0.0149 |
| Obesity I (BMI 30-35) | 1.08 | 1.05 - 1.10 | <0.00001 |
| Obesity II (BMI 35-40) | 1.17 | 1.14 - 1.20 | <0.00001 |
| Obesity III (BMI ≥ 40) | 1.37 | 1.32 - 1.41 | <0.00001 |
| Missing | 0.29 | 0.26 - 0.32 | <0.00001 |
| **Labs**** | | | |
| White blood cell count ≤ 11 | 1.00 | 1.00 - 1.00 | <0.00001 |
| White blood cell count >11 | 1.52 | 1.49 - 1.55 | <0.00001 |
| Hemoglobin ≥ 10 | 0.90 | 0.90 - 0.90 | <0.00001 |
| Hemoglobin <10 | 1.69 | 1.65 - 1.73 | <0.00001 |
| Platelet count ≥ 50 | 1.00 | 1.00 - 1.00 | <0.00001 |
| Platelet count < 50 | 1.75 | 1.72 - 1.79 | <0.00001 |
| White blood cell count ≤ 11 | 1.41 | 1.30 - 1.52 | <0.00001 |
| White blood cell count > 11 | 0.31 | 0.30 - 0.32 | <0.00001 |
| Alanine aminotransferase ≤ 260 | 1.00 | 1.00 - 1.00 | <0.00001 |
| Alanine aminotransferase > 260 | 2.47 | 2.30 - 2.64 | <0.00001 |
| Total bilirubin ≤ 2.4 | 1.06 | 1.06 - 1.07 | <0.00001 |
| Total bilirubin > 2.4 | 2.25 | 2.15 - 2.36 | <0.00001 |
| Albumin ≥ 3.5 | 0.49 | 0.48 - 0.50 | <0.00001 |
| Albumin < 3.5 | 2.29 | 2.25 - 2.34 | <0.00001 |
| Creatinine ≤ 2 | 1.07 | 1.06 - 1.08 | <0.00001 |
| Creatining > 2 | 1.50 | 1.44 - 1.57 | <0.00001 |
| eGFR < 30 | 1.36 | 1.30 - 1.42 | <0.00001 |
| **Treatment***** | | | |
| Chemotherapy | 3.75 | 3.69 - 3.81 | <0.00001 |
| Immunotherapy | 3.07 | 2.98 - 3.15 | <0.00001 |
| Targeted Therapy | 2.05 | 2.01 - 2.10 | <0.00001 |
| Endocrine Therapy | 0.33 | 0.31 - 0.34 | <0.00001 |

**Abbreviations**: RUCA, rural-urban commuting area codes; HIV, human immunodeficiency virus; AIDS, acquired immunodeficiency syndrome; COPD, chronic obstructive pulmonary disease; VTE, venous thromboembolism; ATE, arterial thromboembolism; BMI, body mass index; eGFR, estimated glomerular filtration rate

*Region 1 (Connecticut, Maine, Massachusetts, New Hampshire, Rhode Island, Vermont); Region 2 (New Jersey, New York, Puerto Rico, the Virgin Islands); Region 3 (Delaware, District of Columbia, Maryland, Pennsylvania, Virginia, West Virginia); Region 4 (Alabama, Florida, Georgia, Kentucky, Mississippi, North Carolina, South Carolina, Tennessee); Region 5 (Illinois, Indiana, Michigan, Minnesota, Ohio, Wisconsin); Region 6 (Arkansas, Louisiana, New Mexico, Oklahoma, Texas); Region 7 (Iowa, Kansas, Missouri, Nebraska); Region 8 (Colorado, Montana, North Dakota, South Dakota, Utah, Wyoming); Region 9 (Arizona, California, Hawaii, Nevada, American Samoa, Commonwealth of the Northern Mariana Islands, Federated States of Micronesia, Guam, Marshall Islands, Republic of Palau); Region 10 (Alaska, Idaho, Oregon, Washington)

**Missing lab values were modeled as a separate category and results are not shown

***Time-varying Covariate
